# Genomic analysis of the nitrate-respiring *Sphingopyxis granuli* (formerly *Sphingomonas macrogoltabida*) strain TFA

**DOI:** 10.1186/s12864-016-2411-1

**Published:** 2016-02-04

**Authors:** Inmaculada García-Romero, Antonio J. Pérez-Pulido, Yolanda Elisabet González-Flores, Francisca Reyes-Ramírez, Eduardo Santero, Belén Floriano

**Affiliations:** Centro Andaluz de Biología del Desarrollo, CSIC-Universidad Pablo de Olavide, ES-41013 Seville, Spain

**Keywords:** *Sphingopyxis*, *S. granuli*, *oriC* signature, Genomic islands, Nitrate respiration, Nitrite toxicity, Core genome

## Abstract

**Background:**

Sphingomonads are *Alphaproteobacteria* that belong to the *Sphingomonas*, *Novosphingobium*, *Sphingopyxis* or *Sphingobium* genera, They are physiologically diverse and broadly distributed in nature, playing important roles in oligotrophic environments and in the degradation of recalcitrant polyaromatic compounds, *Sphingopyxis* is a poorly studied genus of which only one representative (*S. alaskensis* RB2256) has been deeply characterized. In this paper we analyze the genomic features of *S. granuli* strain TFA (formerly *Sphingomonas macrogoltabida*) in comparison with the available *Sphingopyxis* sequenced genomes, to describe common characteristics of this genus and to highlight unique characteristics of strain TFA.

**Results:**

The TFA genome has been assembled in a single circular chromosome of 4.7 Mb. Genomic sequence analysis and proteome comparison re-assigned the TFA strain to the *Sphingopyxis* genus and the *S. granuli* species. Some regions of the TFA genome show high similarity (ca. 100 %) to other bacteria and several genomic islands have been detected. Pathways for aromatic compound degradation have been predicted but no growth of TFA has been detected using these as carbon or nitrogen sources. Genes for nitrate respiration have been identified as TFA exclusive. Experimental data on anaerobic growth of TFA using nitrate as a terminal electron acceptor are also provided.

**Conclusions:**

*Sphingopyxis* representatives form a compact phylogenetic group (with the exception of *S. baekryungensis* DSM 16222) that share several characteristics, such as being naturally resistant to streptomycin, having only one ribosomal operon, a low number of prophages and CRISPR sequences, absence of selenoproteins and presence of ectoin and other biosynthesis pathways for secondary metabolites. Moreover, the TFA genome organization shows evidence of the presence of putative integrative and conjugative elements (ICE) responsible for the acquisition of several characteristics by horizontal transfer mechanisms. *Sphingopyxis* representatives have been described as strict aerobes but anaerobic growth using nitrate as a terminal electron acceptor might confer an environmental advantage to the first *S. granuli* strain characterized at genomic level.

**Electronic supplementary material:**

The online version of this article (doi:10.1186/s12864-016-2411-1) contains supplementary material, which is available to authorized users.

## Background

Sphingomonads have been described as a bacterial group within the *Sphingomonadaceae* Family that comprises physiologically diverse α-proteobacteria [1]. Their members are classified into four different genera, *Sphingomonas*, *Novosphingobium*, *Sphingopyxis* and *Sphingobium* [2]. They have attracted attention mainly because of their metabolic diversity, which includes their capacity for xenobiotic degradation as one of the most important characteristics, and their ubiquity, as they have been isolated from many different environments. Some members of this group have been described as oligotrophic bacteria which play an important role in marine environments [3].

Several studies have analyzed the genomic characteristics of sphingomonads to gain insights into their environmental and metabolic adaptations [4–6]. These studies have provided genomic features to describe oligotrophic bacteria [4], have highlighted the diversity in their genome organization [5] and defined genes involved in the quorum sensing metabolism, marine adaptation and bioremediation in the well-known *Novosphingobium* genus [6].

The *Sphingopyxis* genus was first described in 2001 as a group of strictly aerobic and chemo-organotrophic bacteria incapable of nitrate reduction [2]. Abundant partial 16S ribosomal RNA gene sequence information can be found on public databases, which has been used for the ascription of bacterial isolates to the *Sphingopyxis* genus. However, genomic sequence information is available just for seven isolates, of which only two, *Sphingopyxis alaskensis* RB2256 [3, 4] and *Sphingopyxis fribergensis* sp. Kp5.2 [7] have completely assembled genomes. Scaffold genomes are available for *Sphingopyxis* sp. MC1, which was isolated from activated sludge from a waste water treatment plant in Seattle (USA) because its capacity for triclosan removal (unpublished data), and for *S. baekryungensis* DSM 16222, which was isolated from the Yellow Sea in Korea [8] but whose biodegradation capabilities have not been described. Recently, four more strains have been sequenced and also ascribed to the *Sphingopyxis* genus. Strains LC81 and LC363 have been isolated from a limestone formation at −347 m deep in Lechuguilla Cave in New Mexico and described as oligotrophic bacteria [9]; strain MWB1 that comes from a shoreline contaminated by a crude-oil spill in Tae-an, South Korea [10], and *Sphingopyxis* sp. C-1 (released in June 2015 and unpublished), described as a microcystin-degrading bacterium. Despite all this information, only *S. alaskensis* RB2256 has been described in detail and presented as a model of marine oligotrophic bacteria [4, 11].

TFA is a small, rod-shaped, aerobic, streptomycin-resistant and Gram-negative bacterium able to grow on the organic solvent tetralin as the sole carbon and energy source, isolated from mud from the Rhine river [12]. The metabolic pathway for degradation of this aromatic compound has been completely elucidated [13] (and references therein) and the regulation of the expression of the structural and regulatory genes has been characterized [14] (and references therein). This study analyzes the assembled and annotated genomic sequence of strain TFA, which strongly supports its ascription to the *Sphingopyxis* genus, and provides *in silico* and experimental evidence of anaerobic growth using nitrate as an electron acceptor, which has not been previously described for members of this genus. Like other *Sphingopyxis* genus members, TFA shows genomic characteristics described for oligotrophic representatives and horizontal transfer seems to have played an important role in its genome organization.

## Results and discussion

### General features of the TFA genome

The TFA genome was sequenced using a whole-genome shotgun strategy and Roche 454 GS-FLX Titanium pyrosequencing technology. Sequences were assembled *in silico* using a Celera Assembler resulting in 42 contigs. By overlapping PCRs, Southern hybridization and cosmid sequencing, these 42 contigs were assembled into one circular chromosome with 4250 predicted genes which represent 89.47 % of coding sequence. No free plasmids were detected in the assembled sequence. The whole annotated sequence presented in this paper has been deposited in the National Centre for Biotechnology Information (NCBI) under BioProject number PRJNA283604 and GenBank accession number CP012199. General features of the TFA genome are summarized in Table 1. The genome size of *Sphingopyxis* representatives ranges from ca. 3 Mb of *S. baekryungensis* DSM 16222 to ca. 4.9 Mb of *S. fribergensis* Kp5.2. These differences in size could be related to increased environmental versatility in those with larger genomes. The GC content is higher than 60 % in all sequenced *Sphingopyxis* and only two of them, *S. alaskensis* RB2256 and *S. fribergensis* Kp5.2, bear free plasmids (28 and 208 Kb, respectively).

**Table 1:** General genome features of *Sphingopyxis granuli* strain TFA

Characteristic	Value
Genome size (bp)	4,679,853
GC Content (%)	66.2
Coding sequence	4,187,393
Number of predicted genes	4250
Number of predicted protein-coding genes	4190
Predicted proteins with description	3502
Predicted proteins uncharacterized	688
Number of RNA genes	60
tRNA genes	46
rRNA genes	3
ncRNA ^a^	11
Number of protein coding genes in *Sphingopyxis* core	2294
Number of exclusive protein coding genes	479
Other features	
Free plasmids	n.d.
Selenocysteine tRNA and selenoproteins	n.d.

^a^ncRNAs predicted by Infernal software. *n.d*. non-detected

The origin of replication (*oriC* type) has been located, using the web tool Ori-Finder [15], close to *hemE* (SGRAN_0274) as has been predicted for most *Sphingomonadaceae* included in the DoriC database [16]. A comparison of this genomic region in different *Sphingomonadaceae* members revealed conservation of the genetic organization around *oriC* (Additional file 1A). Moreover, several well-conserved putative DnaA binding boxes plus a putative duplex unwinding element (DUE) [17] can be detected by comparison of the intergenic region sequence of *Sphingopyxis* strains and TFA (Additional file 1B). This analysis strongly supports the use of this well-conserved gene cluster to identify the replication origin in α-proteobacteria, as proposed by Brassinga et al. [18]. Accordingly, the predicted origin of replication of *S. alaskensis* RB2256 annotated in the DoriC database should be moved to the corresponding *hemE* region.

Only one ribosomal operon is predicted in TFA genome, which is another general feature in all sequenced genomes of *Sphingopyxis* representatives. Moreover, this is a characteristic already described in bacteria with an oligotrophic life style [4].

All possible codons are used in TFA for protein translation and all can be read with the identified set of tRNAs by a wobble base pair, which is actually required to read the most abundant Arg (CGC) and Tyr (TAT) codons in TFA. No selenocysteine tRNA or putative selenoproteins were annotated. The absence of selenoproteins can be regarded as another feature of *Sphingopyxis* genus extended to all Sphingomonads with sequenced genomes. In fact, according to Zhang et al. [19], the genes for both Sec-decoding and for selenouridine utilization have been found so far in *Paracoccus denitrificans*, an α-proteobacteria belonging to the *Rhodobacteraceae* family.

A total of 4190 protein-encoding genes were predicted in the TFA genome using Prodigal software [20] and further manual curation. Functional annotation of protein-coding genes was performed as described in Methods using the Sma3s program [21], which proved to be highly accurate with bacterial sequences and allows the tracing of the source of each annotation, and manual BLASTp analysis [22].

### Phylogenetic ascription of TFA to the *Sphingopyxis* genus

**Fig. 1:**
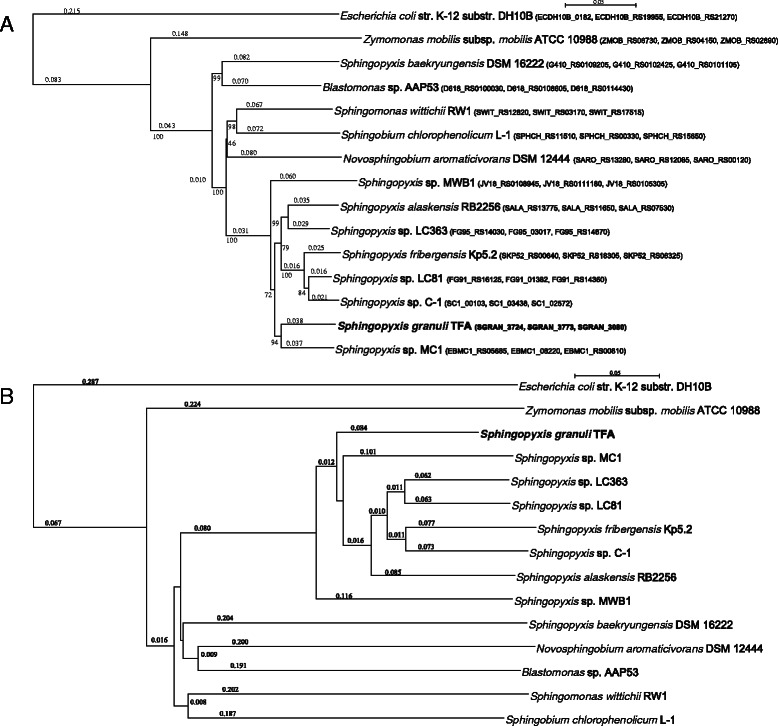
Phylogenetic analysis of the TFA strain. **a** Phylogenetic tree from the concatenated sequences of 16S rRNA, *atpD* and *rpoB. Numbers* under branches correspond to bootstrap percentage values. The locus tags for 16S rRNA, *atpD* and *rpoB* genes, respectively, are indicated in *brackets* for each species. **b** Average Amino acid Identity-based dendogram. *Escherichia coli* str. K-12 substr. DH10B was included as an outgroup in both analysis. *Numbers* above branches represent the branch length

Based on the sequence of an internal 16S rDNA gene fragment, strain TFA was initially ascribed to the *Sphingomonas* genus as a *S. macrogoltabida* species [12, 23]. To validate this result, a new phylogenetic analysis was performed using the complete sequence of the housekeeping genes coding for the 16S ribosomal RNA (SGRAN_3724), the beta subunit of the membrane ATP synthase (*atpD*; SGRAN_3773) and the β subunit of RNA polymerase (*rpoB*; SGRAN_3080). A tree based on ClustalO alignments of the concatenated nucleotide sequences and built with the Neighbor-Joining method clearly shows that strain TFA should be ascribed to the compact group formed by *Sphingopyxis* representatives (Fig. 1a). On the other hand, ascription of *S. baekryungensis* DSM 16222 to the *Sphingopyxis* genus is less obvious, since its position in the phylogenetic tree is in a different branch. Average nucleotide identity (ANI) calculation (Table 2) of *Sphingopyxis* genomes also shows that TFA is very close to this group and that it represents a different species because all ANI values are lower than 0.95–0.96. It is worth noting that the percentage of aligned sequence of *S. baekryungensis* DSM 16222 in this analysis is less than 5 % (Table 2). Moreover, a dendrogram created by clustering pairwise amino acid average identity analysis (AAI) [24] also supports the phylogenetic relationship between TFA and *Sphingopyxis* genus representatives (Fig. 1b and Additional file 2). In this dendrogram, *S. baekryungensis* DSM 16222 again appears in a different branch.

**Table 2:** ANI calculation and percentage of aligned sequence in each comparison in brackets

	*S. alaskensis* RB2256	*S. granuli* TFA	*S. baekryungensis* DSM16222	*Sphingopyxis* sp. LC81	*Sphingopyxis* sp. LC363	*Sphingopyxis* sp. MC1	*Sphingopyxis* sp. MWB1	*S. fribergensis* Kp5.2	*Sphingopyxis* sp. C-1
*S. alaskensis* RB2256		85.50 [48.40]	82.60 [3.08]	86.06 [62.94]	86.77 [65.05]	85.86 [49.73]	84.51 [30.58]	85.82 [60.51]	85.43 [56.82]
*S. granuli* TFA	85.50 [34.70]		83.09 [2.91]	85.13 [38.93]	85.54 [39.84]	85.68 [39.42]	84.45 [20.27]	84.97 [36.06]	84.58 [32.88]
*S. baekryungensis* DSM16222	82.60 [3.47]	83.09 [4.52]		82.92 [3.54]	82.73 [3.30]	82.87 [3.54]	82.71 [3.26]	82.88 [3.38]	82.88 [3.63]
*Sphingopyxis* sp. LC81	86.07 [47.88]	85.13 [41.12]	82.92 [2.38]		87.77 [65.72]	85.13 [38.74]	84.25 [22.36]	88.57 [65.86]	88.00 [67.36]
*Sphingopyxis* sp. LC363	86.76 [52.40]	85.54 [44.41]	82.73 [2.32]	87.77 [69.41]		85.78 [43.00]	84.40 [24.04]	87.47 [68.04]	86.63 [62.07]
*Sphingopyxis* sp. MC1	85.86 [45.18]	85.69 [50.07]	82.87 [2.89]	85.13 [46.41]	85.77 [48.78]		84.56 [25.99]	85.22 [46.34]	84.98 [42.54]
*Sphingopyxis* sp. MWB1	84.50 [32.76]	84.46 [30.26]	82.71 [3.12]	84.25 [31.42]	84.40 [32.02]	84.58 [30.40]		84.23 [28.12]	84.01 [27.56]
*S. fribergensis* Kp5.2	85.81 [40.50]	84.97 [33.68]	82.85 [2.05]	88.56 [58.07]	87.47 [56.73]	85.22 [34.07]	84.22 [17.77]		87.10 [56.85]
*Sphingopyxis* sp. C-1	85.44 [41.48]	84.57 [33.43]	82.88 [2.34]	87.99 [64.45]	86.63 [56.29]	84.97 [33.92]	84.02 [18.73]	87.10 [61.79]	

**Fig. 2:**
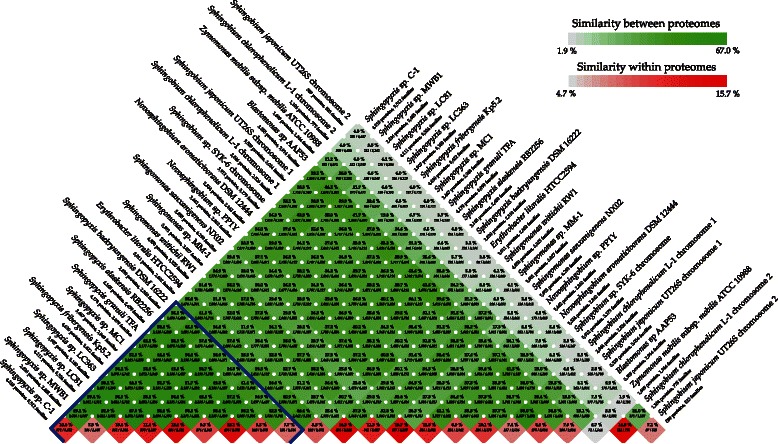
Comparison of proteomes of members of the *Erythrobacteraceae* and *Sphingomonadaceae* families. Comparison was performed by BLAST using the CMG-biotools package parameters. Homology between proteomes and internal homology of each proteome (protein families in paralogous clusters) are represented by different levels of *green* and *red*, respectively. *Sphingopyxis* genus members are under the *blue line*

A more extensive analysis of the complete proteomes of representatives of the *Erythrobacteraceae* and *Sphingomonadaceae* families using the CMG-biotools package [25] also identifies TFA closer to *S. alaskensis* RB2256 and *Sphingopyxis* sp. MC1, and places *S. baekryungensis* DSM 16222 in a more distant position (Fig. 2). Interestingly, TFA and Kp5.2, which have the largest genomes, also show the highest number of paralogs (internal homology) in this analysis. Taken together, all the data support the phylogenetic ascription of TFA to the *Sphingopyxis* genus and provides more evidence for the exclusion of *S. baekryungensis* DSM 16222 from this genus as recently proposed [1].

In an attempt to define the TFA species, a BLASTn analysis of the 16S rRNA gene using the EzTaxon identification service [26] was performed. The most similar species was *Sphingopyxis granuli* strain Kw07(T) with a similarity of 99.65 %. According to all these results, renaming of strain TFA as *Sphingopyxis granuli* strain TFA is proposed and its genome represents the first sequenced genome of this species.

TFA and Kp5.2 strains have been described as naturally resistant to streptomycin. Substitutions of Lysine 42 or Lysine 87 by Arginine in RpsL (protein S12 of the 30S ribosomal subunit) have been described as being responsible for streptomycin resistance in *E. coli* [27] and other bacteria [28]. *Sphingomonadaceae*, *Erythrobacteraceae* and TFA (SGRAN_0292) RpsL proteins show an Arginine in position 87. This fact could explain the natural streptomycin resistance of TFA and Kp5.2 which might be considered a feature of both bacterial families, *Sphingomonadaceae* and *Erythrobacteraceae*.

### *Sphingopyxis* genus core- and pan-genome analysis

Using the CMG-biotools utilities [25] with a cutoff of 70 % coverage and 30 % identity for encoded proteins, a pan- and core-genome plot analysis was performed for all *Sphingopyxis* genomes. The final core genome was found to comprise 1371 gene families and the pan genome contains 6955 gene families. More than half of the TFA protein coding genes (54.7 %; 2294 genes) are included in the core genome indicating a high genetic homogeneity in the *Sphingopyxis* group. A classification of TFA genes belonging to the *Sphingopyxis* core genome in cluster of orthologous groups of proteins (COG) categories (see Additional file 2) shows that lipid transport and metabolism (category I) are highly represented. On the contrary, cell motility (category N) and defense mechanisms (category V) have the lowest number of core genes. This result is in agreement with the genomic features described by Lauro et al. [4] for an oligotrophic lifestyle. In this core genome there are genes involved in copper resistance and genes encoding heavy metal and multidrug efflux pumps. Interestingly, tetralin degradation proteins, except ThnA4, ThnY and ThnM, are also part of this core genome even though some of the corresponding *thn* genes have been included in one TFA genomic island (see below) and their nucleotide sequences are not similar to any gene present in the database.

479 exclusive proteins were detected in TFA proteome. Proteins encoded by the *narUGHJI* operon and the divergently transcribed *ftrB* gene seem to be absent in other *Sphingopyxis* strains. The acquisition of these *nar* genes and the capability of nitrate respiration (see below) are probably related to the characteristics of the TFA environmental niche. Additionally, 38.4 % of the exclusive TFA proteins have unknown functions, which might represent interesting new activities to be studied.

### TFA genome organization: evidence of horizontal transfer, genomic islands and prophage analysis

**Fig. 3:**
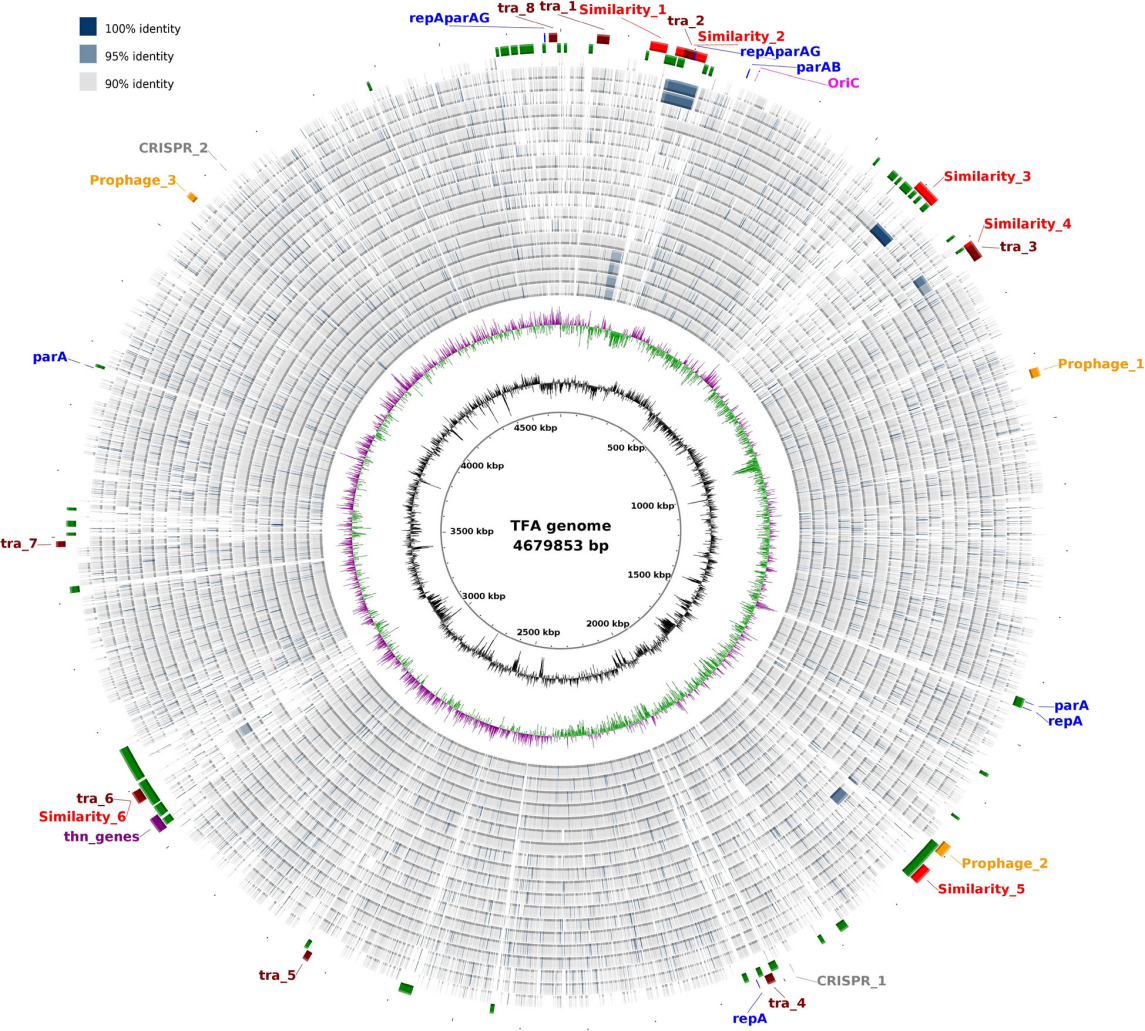
Circular representation of the TFA genome and comparison with other α-proteobacteria. The location of the *oriC* is highlighted in *pink*. Comparison between the genomes of TFA and α-proteobacteria representatives at nucleotide level was performed using BLAST Ring Image Generator (BRIG). From inner to outer ring: (1) GC Skew; (2) GC Content; (3–21) BLASTn comparison between the TFA genome and *Sphingopyxis* sp. MC1, *Sphingopyxis alaskensis* RB2256, *Sphingopyxis fribergensis* Kp5.2, *Sphingopyxis* sp. LC363, *Sphingopyxis* sp. LC81, *Sphingopyxis* sp. MWB1, *Sphingopyxis* sp. C-1, *Sphingopyxis baekryungensis* DSM 1622, *Sphingomonas wittichii* RW1, *Novosphingobium aromaticivorans* DSM 12444, *Novosphingobium* sp. PP1Y, *Erythrobacter litoralis*, *Blastomonas* sp. AAP53, *Sphingobium* sp. SYK-6, *Sphingobium japonicum* UT26, *Sphingobium chlorophenolicum* L-1, *Oligotropha carboxidovorans* OM4, *Oligotropha carboxidovorans* OM5 and *Zymomonas mobilis* genomes; (22) Genomic Islands predicted by IslandViewer 3 by any method (*green*); (23) Features: Clusters of *tra*/*trb* genes (tra_1 to 8, in *brown*); Similar regions to other α-proteobacteria (Similarity_1 to 6, in *red*); Plasmid replication and partition genes (*blue*); Predicted prophages (Prophage_1 to 3, in *yellow*); Predicted CRISPR (CRISPR_1 and 2, in *gray*) and *thn* genes (in *purple*). Accession numbers of the genomes can be found in Additional file 3

A comparison between TFA genome and α-proteobacteria representatives at nucleotide level was performed using BLAST Ring Image Generator (BRIG) [29] (Fig. 3). Accession numbers of the genomes are in Additional file 3. Six regions with a high percentage of sequence identity with some of the selected α-proteobacteria were detected (see red ring in Fig. 3, Table 3 and Additional file 4). Region 1 is 95 % identical to regions in the *Sphingopyxis sp*. MC1, *S. alaskensis* RB2256, *S. fribergensis* Kp5.2 and *Sphingopyxis* sp. LC363 chromosomes and contains genes mainly involved in resistance to heavy metals. Region 2 is 97 % identical to a region of the *Oligrotropha carboxidovorans* OM4 chromosome and contains genes involved in potassium transport, plasmid conjugation, replication and partition, DNA metabolism and many uncharacterized proteins. The presence of plasmid related proteins in this region (see below) could indicate the horizontal transfer of this fragment by conjugation in both, TFA and OM4. Regions 3 and 4 show 99 and 96 % identity, respectively, to regions of the *Sphingobium chlorophenolicum* L1 chromosome. Gene products in region 3 are proteins involved in oxygen sensing and stress response (SGRAN_0579, SGRAN_0580, SGRAN_0582 and SGRAN_0584). Many genes in Region 4 encode conjugative plasmid transfer proteins. Region 5 is 97 % identical to a region of the *Erythrobacter litoralis* HTCC2594 chromosome and contains genes coding for dehydrogenases, di- and mono-oxygenases, hydrolase and transport systems related to drug resistance. Region 6 is very similar (94 % of identity) to genes related to the conjugation process that are also present in the *Novosphingobium* sp. strain PP1Y chromosome.+

**Table 3:** Regions of TFA chromosome with high identity to other bacteria

Region	Start	End	Size (nucleotides)	Similar to	Proteins involved in
1	134,249	159,746	25,497	*Sphingopyxis sp.* MC1 *S. alaskensis* RB2256 *S. fribergensis* Kp5.2 *Sphingopyxis* sp. LC363	Heavy metal resistance
2	173,796	220,082	46,286	*O. carboxidovorans* OM4	K transport, Plasmid replication, DNA metabolism
3	590,566	628,642	38,076	*Sphingobium chlorophenolicum* L1	Oxygen sensing and stress response
4	707,273	722,772	15,499	*Sphingobium chlorophenolicum* L1	Conjugative plasmid transference
5	1,728,811	1,756,443	27,632	*Erythrobacter litoralis* HTCC2594	Hydrolysis, oxygenation, drug resistance
6	3,076,722	3,095,970	19,248	*Novosphingobium* sp. strain PP1Y	Conjugative plasmid transference

Additionally, large regions of the TFA genome have been predicted as Genomic Islands (GIs) using the interface IslandViewer 3 [30] which uses two different methods for prediction, SIGI-HMM and IslandPath. Genes in each GI are listed in Additional file 5. All GIs are indicated in Fig. 3 (green circle) and those predicted by both methods are shown in Table 4. The larger GI (ca 69 kb) contains two clusters of genes coding for proteins involved in biodegradation processes separated by transposase and integrase genes (GI 2 in Table 4). It also contains the region highly similar to the *Erythrobacter litoralis* HTCC2594 chromosome (region 5), which strongly supports its foreign origin. Other large GIs predicted by both methods (GIs 5 and 6) encode proteins related to heavy metal pumps, conjugative transference of plasmids and phage-related proteins. GIs 41 to 47, predicted only by SIGI-HMM, code for proteins involved in copper, mercury and arsenic resistance and many efflux pumps. These regions include genes involved in plasmid replication and partition and conjugation, thus suggesting that they were in a plasmid that integrated into the TFA chromosome. Interestingly, most of the tetralin degradation genes (*thnCA3A4RY*, *thnB*, *thnGHIJKLM*) are also located in SIGI-HMM-predicted GIs (GIs 29 and 30). Intriguingly, a search of DNA sequences similar to TFA *thn* genes using BLASTn with default parameters (not shown) gave no positive results, thus the origin of these genes remains unknown.

**Table 4:** Overlapping Genomic islands predicted by IslandViewer 3 methods

by IslandPath-DIMOB				by SIGI-HMM				Proteins
GI number	Start	End	Size (nucleotides)	GI number	Start	End	Size (nucleotides)	
2	1,691,609	1,760,539	68,930	19	1,691,609	1,722,457	30,848	Biodegradation pathways
				21	1,734,079	1,761,611	27,532	Biodegradation pathways (includes similar region 5)
				22	1,878,882	1,891,309	12,427	Phage proteins (integrases)
4	2,001,817	2,012,061	10,244	24	2,000,239	2,009,439	9200	Uncharacterized proteins
5	3,066,560	3,103,335	36,775	31	3,063,300	3,079,834	16,534	Uncharacterized, heavy metal pumps, phage related and conjugal transfer of plasmids
				32	3,093,114	3,102,991	9877	
6	3,109,158	3,161,042	51,884	33	3,107,612	3,127,918	20,306	
				34	3,128,726	3,138,976	10,250	
				35	3,141,481	3,162,313	20,832	

Many sphingomonads bear large plasmids that contribute to their metabolic diversity and resistance to toxic metals [31]. Free plasmids have not been detected in TFA using different methods of lysis and pulse-field gel electrophoresis (see Methods). Moreover, no evidence of free plasmids was found during assembly of the genomic sequence. However, genes encoding proteins involved in replication initiation (Rep) and plasmid partition (Par) can be identified in different locations of the TFA genome (Fig. 3, in blue). One putative *repAparAG* operon (SGRAN_0191, SGRAN_0190 and SGRAN_0189) is located in the similar Region 2 (see above) and it is close to an extra *parAB* operon lacking the *repA* gene (SGRAN_0260 and SGRAN_0259). A second *repAparAG* operon (SGRAN_4224, SGRAN_4225 and SGRAN_4226) is at the SIGI-HMM-predicted GI 46, very close to the large GIs 43, 44 and 45 which bear metal resistance genes. The rest of the *repA*- or *parA*-like encoding genes are scattered in the chromosome. All TFA RepA and ParA proteins detected belong to the RPA (Pfam 10134) and CbiA (Pfam 01656) superfamilies, respectively.

Besides plasmid replication proteins, a high number of genes encoding proteins involved in plasmid conjugation (*tra/trb* genes), which grouped in eight clusters (Fig. 3, in brown), have been annotated. Some of these clusters overlap with regions highly similar to other bacteria and/or predicted genomic islands, supporting the role of horizontal transfer mechanisms in DNA incorporation into the TFA genome. Although more data should be provided, it is tempting to consider some of these regions as integrative and conjugative elements (ICE) with an important role in the final structure of the TFA genome [32].

The annotation of phage-related genes in the TFA genome might indicate the presence of prophages. Three regions were identified as questionable prophages according to the completeness score given by PHAST (PHAge Search Tool) software [33], (Fig. 3, in yellow). However, *att* sites are only in Prophage_2, which overlaps with the largest predicted GI. Using the same tool, three prophages have been predicted in *S. alaskensis* RB2256, one intact (score above 90), one incomplete (score less than 60) and one questionable (score between 60 and 90), while in *S. fribergensis* Kp5.2 only one incomplete and one questionable were detected (not shown).

Finally, only two CRISPR-related sequences were found in the TFA genome using the CRISPfinder application (Fig. 3, in gray) [34]. CRISPR_1 region, defined as a confirmed CRISPR, is located ca. 300 Kb apart from predicted prophage_2. No Cas-related proteins were found in TFA with this software although one CRISPR-associated Cas1 encoding gene was annotated using Sma3a (SGRAN_3181), which is an exclusive gene of TFA. Using the same tool, only questionable CRISPR sequences were found in the *S. fribergensis* Kp5.2, *Sphingopyxis* sp. LC,363 (contig 45), *Sphingopyxis* MC1 (contig 5), and *Sphingopyxis* MWB1 (contig 2) genomes, and no CRISPRs were predicted in the *S. alaskensis* RB2256, *S. baekryungensis* DSM 16222, or *Sphingopyxis* sp. LC81 genomes.

A low number of both prophages and CRISPR regions are features already defined for oligotrophic bacteria [4] that might also be considered as particularities of the *Sphingopyxis* genus members.

### Prediction of pathways for aromatic compound degradation and secondary metabolites biosynthesis

TFA was initially isolated because of its ability to grow on tetralin (a toxic compound with one aromatic and one alicyclic ring that share two carbon atoms). Only three *Sphingopyxis* are included and analyzed in BioCyc, a pathway/genome database [35], and just a few incomplete pathways for aromatic degradation have been predicted. However, as described for other *Sphingomonadaceae* members, a high number of genes putatively involved in aromatic compound degradation pathways have been annotated in the TFA genome. The predicted metabolic map of TFA using Pathway-Tools [36] shows putative degradation pathways for several aromatic compounds but with important gaps in most of them. Almost complete pathways were predicted for 3-phenylpropanoate, 2-nitrobenzoic and anthranilic acid degradation in which 2-oxopentenoate is a common intermediate (see Additional file 6). A cluster of genes (SGRAN_1577 to SGRAN_1583), flanked by putative insertion elements, are predicted to be involved in 3-phenylpropionate degradation through extradiol cleavage. However, Pathway-Tools detected two gaps, one corresponding to the extradiol cleavage of the aromatic ring and another to the hydrolysis of the resulting linear compound (see Additional file 5). Manual inspection of adjacent genes shows that genes SGRAN_1575 and SGRAN_1576 can encode the alpha and beta subunits of a putative type II extradiol dioxygenase, respectively. Moreover, the product of SGRAN_1584, annotated as a 2-hydroxy-6-oxo-2,4-heptadienoate hydrolase, could catalyze the hydrolytic reaction. Thus, it seems that the 3-phenylpropanoate degradation pathway is complete. Proteins encoded by a second *mhp* operon (SGRAN_1420 to 1422) could also catalyze 2-oxo-pent-4-enoate conversion to acetyl-CoA. Besides, enzymes involved in tetralin degradation (Thn) were predicted to be capable of catalyzing some steps of the pathway (see Additional file 6).

For 2-nitrobenzoic and anthranilic acids, Pathway-Tools predicted a complete pathway from the non-commercial intermediate 3-hydroxyanthranilate. Most of the genes involved (*nba* and *amn* genes) (SGRAN_3549 to SGRAN_3552) are contiguous in the TFA genome and could be part of the same operon. Several gaps have also been found in those predicted pathways (see Additional file 6). For anthranilate degradation, an initial monooxygenation of this compound is needed but no enzyme has been found by Pathway-Tools. However, several putative monooxygenases (e.g., cytochrome P450) are annotated in the TFA genome that could catalyze this reaction. On the contrary, neither nitrobenzoate nitroreductase nor 2-hydroxylaminobenzoate mutase, needed for the first steps in the 2-nitrobenzoic acid biodegradation pathway, was annotated in the TFA genome.

Growth of TFA using any of those compounds as carbon and energy sources or as a nitrogen source in the case of 2-nitrobenzoic or anthranilic acids, has been negative. In an attempt to increase the possibility of gratuitous induction of the genes, tetralin-grown cells were unsuccessfully used as an inoculum. Thus, a plausible explanation for the lack of growth is that the annotated genes have different substrate specificity and represent an example of erroneous sequence-based assignation to degradation pathways. Alternatively, they might be a reminiscence of a partial horizontal transfer of genes that are not transcribed. It is also possible that those genes are expressed only in the presence of certain intermediates produced by other bacteria, forming a consortium in the same niche, for commensal biodegradation processes.

Using the platform for antibiotics and secondary metabolite search antiSMASH (Antibiotics and Secondary Metabolites Analysis Shell) [37], clusters of genes involved in the biosynthesis of ectoin (an osmoprotectant), terpene (which could be related to the pinkish color of some *Sphingopyxis*) and homoserine lactone (involved in cell-to-cell communication) were predicted in most *Sphingopyxis* members. However, identification of ectoin biosynthetic genes failed in *S. baekryungensis* DSM16222 and *Sphingopyxis* sp. LC81. Homoserine lactone biosynthetic genes were not found in *S. baekryungensis* DSM16222 or *Sphingopyxis* sp. MWB1. Finally, TFA lacks terpene biosynthesis genes found in the other *Sphingopyxis* genomes, which could explain the yellowish color of this strain.

### Nitrate reduction and anaerobic growth

Although *Sphingopyxis* have been considered incapable of nitrate reduction, this capacity has been described for *S. granuli* Kw07 [38] and *S. baekryungensis* [8].

**Fig. 4:**
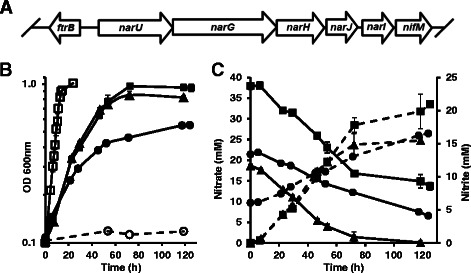
Nitrate respiration in TFA. Genetic organization of TFA *nar* genes (**a**). Anaerobic growth of TFA, measured by OD increase (**b**), in rich medium plus nitrate 20 mM (*open squares*), or in minimal medium in the presence of nitrate 40 mM (*squares*), 20 mM (*triangles*) or nitrate 20 mM plus nitrite 6 mM (*circles*). No growth was detected when nitrate was omitted from the growth medium (*open circles*). Nitrate consumption and nitrite production (**c**, *solid* and *dotted lines*, respectively) in the presence of nitrate 40 mM (*squares*), nitrate 20 mM (*triangles*) or nitrate 20 mM plus nitrite 6 mM (*circles*)

TFA does not grow using nitrates or nitrites as nitrogen sources (see Additional file 7) and assimilatory nitrate or nitrite reductases have not been identified in its genome. However, a cluster of genes coding for proteins involved in nitrate respiration has been found (Fig. 4a). Gene *narU* (SGRAN_3845) is annotated as coding for a nitrate/nitrite transporter while the *narG* (SGRAN_3846), *narH* (SGRAN_3847) and *narI* (SGRAN_3849) genes encode, respectively, alpha, beta and gamma subunits of a respiratory nitrate reductase. Forming part of the same operon, gene *narJ* (SGRAN_3848) codes for a chaperone involved in the assembly of the molybdenum cofactor. Expression of this *nar* operon might be regulated by a transcriptional regulator, transcribed in the opposite direction, similar to FtrB (SGRAN_3844), an activator upregulated in response to oxygen limitation in *Caulobacter crescentus* [39]. Thus, this cluster of genes could be responsible for the anaerobic utilization of nitrate as an electron acceptor in TFA as seen in other nitrate-reducing bacteria. Although TFA also encodes a quinol-linked nitric oxide reductase (SGRAN_3801), it lacks the genes necessary for a complete denitrification pathway like *nirS* or *nirK*, coding, respectively, for the cytochrome cd1 and the NirK copper-containing enzyme for nitrite reduction to nitric oxide and *nosZ* for nitrous oxide reduction to N_2_.

Anaerobic growth has not been described for any member of the *Sphingopyxis* genus before. Anaerobic growth of TFA in rich medium in the presence of nitrate 20 mM was monitored by optical density (OD) increase (Fig. 4b, open squares). Anaerobic growth was also tested in minimal medium supplemented with nitrate 20 mM as electron acceptor and ammonium as nitrogen source (Fig. 4b, triangles). Doubling times of 4.06 ± 0.5 and 14 ± 0.3 h were calculated for TFA anaerobic growth in rich and minimal media, respectively. No growth was obtained when nitrate was omitted (Fig. 4b, open circles). As a consequence of nitrate reduction, nitrite accumulates in the growth medium resulting in a conversion rate of 1:1, which indicates that nitrite cannot be reduced (Fig. 4c, dotted lines). Nitrite concentrations higher than 20 mM in the medium seem to be toxic to TFA as no significant differences in growth, nitrite accumulation or nitrate consumption was observed when nitrate concentration was increased to 40 mM (Fig. 4b and c). Moreover, TFA anaerobic growth with nitrate 20 mM is affected by the presence of nitrite 6 mM added to the medium, thus resulting in a half yield in OD (Fig. 4b, solid circles). In this case, nitrate is just partially consumed, until nitrite accumulates to toxic levels (Fig. 4c, circles in solid and dotted lines, respectively).

The lack of *nir* genes or the *nrf* (formate-dependent nitrite to ammonia) operon could explain the accumulation of nitrite in the medium. Nitrite has been described as toxic for bacteria but TFA resistance to 20 mM nitrite might be due to the presence of a nitrate/nitrite antiporter (NarU; SGRAN_3845) similar to NarK in *E. coli*, which is involved in a nitrite-extrusion system during anaerobic nitrate respiration [40]. It is also possible that in environmental conditions, nitrite does not accumulate due to the activity of other bacteria thus allowing a higher TFA cell density respiring nitrate. Genes coding for dissimilatory nitrate reduction have been described in other *Sphingomonadaceae* and its implication in aerobic dissimilatory reduction of nitrate has been suggested [5]. However, this aerobic activity has been linked to the expression of a periplasmic nitrate reductase [41] different from the membrane-bound enzyme of anaerobic denitrifiers. Our data demonstrate that, in agreement with the annotated genes in its genome, TFA is capable of anaerobic or microaerobic growth using nitrate as the electron acceptor, although nitrite cannot be further respired. This ability should represent an advantage in low oxygen concentration environments like the mud of the Rhine river or in environments with frequent transition between oxic and anoxic conditions. The latter has been proposed as the best selective criterion for isolating aerobic denitrifiers [41].

## Conclusions

The data presented in this paper show that the *Sphingopyxis* genus is a compact group in which the affiliation of *S. baekryungensis* DSM 16222 should be revised. Additionally, we have found that several genomic features, previously described for oligotrophic bacteria, are shared by all *Sphingopyxis* despite not all of them having been described as oligotrophs. We have defined a specific gene organization and a consensus sequence for DnaA binding boxes for *oriC* identification in *Sphingopyxis* genomes*.* A specific mutation, detected in all *Sphingopyxis* RpsL proteins, explains natural streptomycin resistance of TFA and Kp5.2, a characteristic that should be extensive to all *Sphingopyxis*. TFA has acquired important functions, such as resistance to some heavy metals, by horizontal transfer. Finally, nitrate respiration is an exclusive characteristic of the first *S. granuli* strain characterized at genomic level.

## Methods

### Whole-genome sequencing and assembly

Genomic DNA from the TFA strain was prepared by using the Wizard Genomic DNA purification system (Promega). Its quality and quantity was assessed with Quant-It-Picogreen (Invitrogen) and a Nanodrop ND-1000 Spectrophotometer (Thermo Fisher Scientific). 12.5 μg of purified DNA was sent to the company LifeSequencing (http://www.lifesequencing.com/, Valencia) for sequencing using a 454 GS-FLX platform. Assembly of the raw sequences by a Celera assembler resulted in 42 contigs. Final assembly was done by searching each contig end against the UniProt database using BLASTx [42], detecting truncated proteins (bridge proteins), which match in more than one contig end and designing primers at the end of those contigs to fill the gaps [43]. Some additional steps were needed to close the genome, consisting of Southern Blot assays against a TFA genomic DNA library using probes designed from contig ends, and then sequencing positive cosmids in order to find contiguous ends.

### Data submission

The genome sequence and its annotations have been deposited in GenBank under the accession number CP012199. The phylogenetic data from the concatenated sequences of 16S rRNA, *atpD* and *rpoB* genes have been submitted to TreeBASE and are available for download at https://treebase.org/treebase-web/search/study/summary.html?id=18711.

### Protein-coding gene prediction and functional annotation

After the closure of the TFA genome, Open Reading Frames (ORFs) for protein-coding genes were searched for using Prodigal v1.20 Analysis Server, a microbial gene finder which has already proved its accuracy and specificity [20]. Next, the predicted proteins were functionally annotated using a modified version of the Sma3s program [21], which has demonstrated its high accuracy with bacterial sequences, allows the tracing of the source of each annotation and initially tries to discover the query sequences in the annotated database. It uses the UniProt database to assign gene names, descriptions and EC (Enzyme Commission) numbers to the query sequences and adds GO terms, UniProt keywords and pathways, InterPro motifs and domains and interaction from the IntAct database, all of which will be useful for functional characterization. We performed a modified protocol to obtain high sensitivity and quality by rewarding manually curated annotations. Thus, the FASTA file with the predicted amino acid sequences was used as input for 6 executions of the Sma3s program: 3 against the Bacteria Swiss-Prot database (manually curated) and another 3 against Bacteria TrEMBL database (automatically annotated), using the three Sma3s annotator modules independently in both groups: A1 for searching itself, A2 for searching orthologs, and A3 for a more exhaustive search. All the results were merged to enrich the final set of annotations, giving priority to the Swiss-Prot annotations and avoiding uncharacterized annotations and non-informative gene names. A total of 4086 proteins from the 4298 predicted by Prodigal were annotated with Sma3s (95.06 %). An additional automatic annotation was performed by WebMGA [44] to assign COG categories to each protein. To increase the number of annotated genes, we performed a manual search of similar proteins in the non-redundant database in the NCBI BLASTp web tool and assigning the annotation of the best hit fulfilling a threshold (identity >30 %, query coverage >70 %, e-value ≤1e-05). A total of 120 proteins from the 212 structurally predicted by Prodigal were annotated in this step. After accepting all changes recommended by the Discrepancy Report, generated by the NCBI submission tool tbl2asn (http://www.ncbi.nlm.nih.gov/Genbank/tbl2asn2.html), a total of 4190 protein encoding genes were finally annotated (97.49 % of those predicted by Prodigal).

### In silico ncRNA identification

The tRNA gene prediction was exhaustively performed using three programs: ARAGORN [45], tRNAscan-SE [46] and tRNAfinder [47]. In order to identify the initiator tRNA-Met, the TFAM program, an online tRNA function classifier [48], was used. Genes for rRNA genes were identified by RNAmmer [49]. Manual inspection of the RNAmmer-predicted 16S rDNA gene detected a truncated anti-Shine-Dalgarno sequence. To prevent erroneous annotation, Infernal 1.1 software [50] was used to predict the most accurate 16S rDNA sequence in TFA, which has been included in the final annotation.

Finally, a search of noncoding RNAs was performed using the cmsearch option of Infernal version 1.1 against the pre-calibrated covariance models of the Rfam database release 12.0, keeping the default settings and discarding predictions with an e-value ≤1e-05.

### Comparative genomic approaches

Comparisons between genomic sequences were carried out with BRIG (BLAST Ring Image Generator) [29], based on BLASTn of the complete genomes. Accession numbers of the sequences used in comparisons are available in Additional file 3.

To find the TFA *oriC* sequence, the genomic sequence together with the annotation file in .ptt format were used to look for the *oriC* using the web program Ori-Finder (http://tubic.tju.edu.cn/Ori-Finder) [15], with default parameters using *E. coli* DnaA boxes as a template.

### Phylogenetic analysis

The ascription of TFA to the *Sphingopyxis granuli* species was carried out using the EzTaxon identification service (http://www.ezbiocloud.net/eztaxon) [26], comparing the TFA 16S rRNA gene against its extensive 16S rDNA database of cultured and uncultured bacteria (eztaxon-e database).

The average nucleotide identity (ANI) of the TFA genome compared with other bacteria genomes was calculated using the JSpeciesWS server (http://jspecies.ribohost.com/jspeciesws/) [51], and only the result of the MUMmer based calculation was selected to be shown.

The average amino acid identity (AAI) was calculated for a representative of each *Sphingomonadaceae* genus with a sequenced genome (completely assembly or not), except for the *Sphingopyxis* genus where all representatives were selected. The AAI was calculated by comparing all protein-coding sequences from a genome against the entire proteome from another and repeating the same action with all the proteomes. The threshold was the same as that used for the proteome matrix, as previously recommended [24], but in this case we used a modified version of the Sma3s module 2 (based on BLAST Reciprocal Best Hits), which gives only the list of identity percentages obtained in each comparison. Then, a distance matrix was constructed to generate a dendrogram applying the fneighbor program of the embassy-phylip package, using the Neighbor-Joining method, and visualized with SeaView [52].

The concatenated nucleotide sequences from 16S rRNA, *atpD* (ATP synthase subunit beta) and *rpoB* (DNA-directed RNA polymerase subunit beta) genes were aligned using ClustalO and used to build a phylogeny, selecting the Neighbor-Joining algorithm in the SeaView program. A bootstrap test of 1000 replicates was applied to validate the phylogeny.

The construction of the pairwise comparison matrix of *Sphingomonadales* proteomes and the pan-core genome plot of *Sphingopyxis* representatives were performed using the matrix and pancoreplot scripts of the CMG-biotools package, in both cases considering a threshold of 30 % identity and 70 % coverage for the longest protein in the BLASTp. TFA specific genes within the *Sphingopyxis* genus were extracted from the pan-core plot data with the pancoreplot_subsets program.

### Genomic islands, prophages and CRISPRs detection

A search was made for genomic islands (GIs) in the TFA genome using the web server IslandViewer3 (http://www.pathogenomics.sfu.ca/islandviewer/browse/) [30], which is an integrated interface for computational identification and visualization of genomic islands. It was executed with default parameters using the GI prediction method SIGI-HMM [53], which is based on codon usage, and IslandPath-DIMOB [54], which considers dinucleotide sequence composition bias and the presence of mobility genes. The GI prediction thus obtained was integrated into the Brig image using the coordinates and the table with genes included in the GI predicted by each method is shown in Additional file 4. Furthermore, prophage sequences were detected with PHAST (PHAge Search Tool) (http://phast.wishartlab.com/) [33], also with default parameters and, finally, putative CRISPR element identification and CAS proteins detection were performed using CRISPRFinder (http://crispr.u-psud.fr/Server/) [34].

### Gene network/pathway analysis

A species-specific Pathway/Genome Database (PGDB) was built using the PathoLogic component of Pathway Tools software [36]. The reactions were inferred from the Gene Ontology codes and the EC numbers were assigned to each protein during the annotation procedure. A complete metabolic map was generated and the pathways involved in aromatic compound degradation were specifically searched.

The capability of *Sphingopyxis* members to synthesize antibiotics and secondary metabolites was analyzed by the web version of antiSMASH (Antibiotics and Secondary Metabolites Analysis Shell; http://antismash.secondarymetabolites.org/) [37] using the nucleotide sequences in FASTA format as input and with default parameters.

### TFA growth conditions

TFA was routinely grown in an aerobic rich medium MML [12] at 30 °C. For anaerobic growth, TFA was pre-cultured in an aerobic rich medium to an OD_600_ of 1. Aliquots were then transferred into standing stoppered bottles filled to the top in rich or MM minimal media [55] containing β-hidroxybutyrate 40 mM as a carbon source to an initial OD_600_ of 0.1. Sodium nitrate 20 or 40 mM was added as a final electron acceptor when required. To assay nitrite toxicity, an initial concentration of 6 mM of sodium nitrite was added to the anaerobic culture. Streptomycin 50 μg/ml was used as antibiotic selection. Growth was determined by measuring OD 600 nm.

### Plasmid isolation methods

Plasmid isolation from exponentially growing TFA cells in MML was carried out by the in-gel cell lysis method previously described [56] and by the lytic method for pulse field electrophoresis [57].

### Nitrite and nitrate determination

Nitrite and nitrate concentration in the growth media were measured as described previously [58]. Briefly, for nitrite determination, cultures were centrifuged and filtered. One millilitre dilutions of the resulting medium were mixed with 1 ml of a saturated solution of sulfanilic acid prepared in 20 % (*v/v*) of HCl and 1 ml of aqueous solution of 0.2 % (*w/v*) N-(1-naphthyl)-ethylendiamine dihydrochloride. After 15 min at room temperature, absorbance at 540 nm was measured and nitrite concentration determined using a standard curve prepared with sodium nitrite. For nitrate determination cultures were centrifuged, filtered and the nitrite completely removed from the medium by the addition of amidosulfonic acid to a final concentration of 1 % (*w/v*) and overnight incubation. Dilutions of the resulting nitrite-free medium were mixed with an H_2_SO_4_:H_3_PO_4_ solution (1:1 in volume) and a fresh solution of 2, 6-dimethylphenol (0.12 %, *w/v*, in concentrated acetic acid). After 20 min at room temperature, absorbance at 324 nmwas measured and the nitrate concentration determined using a standard curve prepared with sodium nitrate.

### Availability of supporting data

All the supporting data are included as additional files.

## Additional files

Additional file 1:
**Analysis of the** ***oriC*** **genomic region. A. Genetic organization around the predicted** ***oriC*** **region in several** ***Sphingomonadaceae*** **representatives.** The *oriC* region (line in purple) is predicted between *yqfL* (in black) and *hemE* (in red). Two other genes, encoding a membrane protein (in blue) and the Rho factor (in green), are also conserved. Non-conserved genes are in gray. B. Clustal alignment of the predicted *oriC* sequences of *Sphingopyxis* strains with sequenced genomes. Recognized DnaA binding boxes, predicted by OriC Finder, are indicated in gray and the putative duplex unwinding element (DUE) is highlighted in black. (PDF 70 kb) Additional file 2:
**Classification proteins encoded by the** ***Sphingopyxis*** **genus core genome in COG categories.** COG categories are A, RNA processing and modification; C, Energy production and conversion; D, Cell cycle control, cell division, chromosome partitioning; E, Amino acid transport and metabolism; F, Nucleotide transport and metabolism; G, Carbohydrate transport and metabolism; H, Coenzyme transport and metabolism; I, Lipid transport and metabolism; J, Translation, ribosomal structure and biogenesis; K, Transcription; L, Replication, recombination and repair; M, Cell wall/membrane/envelope biogenesis; N, Cell motility; O, Posttranslational modification, protein turnover, chaperones; P, Inorganic ion transport and metabolism; Q, Secondary metabolites biosynthesis, transport and catabolism; R, General function prediction only; S, Function unknown; T, Signal transduction mechanisms; U, Intracellular trafficking, secretion, and vesicular transport; V, Defense mechanisms. (PDF 63 kb) Additional file 3:
**Accession numbers of bacterial genomes used in this paper.** (DOCX 16 kb) Additional file 4:
**Genes present in TFA genomic regions highly similar to other α-proteobacteria genomes.** (PDF 83 kb) Additional file 5:
**Genes present in predicted Genomic Islands (GI) in the TFA genome.** Highlighted in red and bold are those genes predicted in GIs by both SIGI-HMM and IslandPath methods. (PDF 48 kb) Additional file 6:
**Pathway Tools biodegradation pathways predicted in TFA.** ID of genes involved in each reaction is shown in red. (PDF 53 kb) Additional file 7:
**Aerobic growth of TFA using different nitrogen sources: ammonium 15 mM (triangles), nitrate 20 mM (squares), nitrite 10 mM (circles), nitrite 5 mM (diamonds) and without nitrogen source added (cross). **(PDF 28 kb)

## Abbreviations

AAI: average identity analysis
ANI: average nucleotide identity
antiSMASH: antibiotics and secondary metabolites analysis shell
BRIG: BLAST ring image generator
COG: cluster of orthologous groups of proteins
DUE: duplex unwinding element
EC: enzyme commission
GI: genomic island
ICE: integrative and conjugative elements
NCBI: National Centre for Biotechnology Information
OD: optical density
PGDB: pathway/genome database
PHAST: PHAge search tool

## References

[1] Glaeser S, Kämpfer P, Rosenberg E, DeLong E, Lory S, Stackebrandt E, Thompson F (2014). The family sphingomonadaceae. The prokaryotes SE–302.

[2] Takeuchi M, Hamana K, Hiraishi A (2001). Proposal of the genus *Sphingomonas* sensu stricto and three new genera, *Sphingobium*, *Novosphingobium* and *Sphingopyxis*, on the basis of phylogenetic and chemotaxonomic analyses. Int J Syst Evol Microbiol.

[3] Eguchi M, Ostrowski M, Fegatella F, Bowman J, Nichols D, Nishino T (2001). *Sphingomonas alaskensis* strain AFO1, an abundant oligotrophic ultramicrobacterium from the North Pacific. Appl Environ Microbiol.

[4] Lauro FM, McDougald D, Thomas T, Williams TJ, Egan S, Rice S (2009). The genomic basis of trophic strategy in marine bacteria. Proc Natl Acad Sci U S A.

[5] Aylward FO, McDonald BR, Adams SM, Valenzuela A, Schmidt RA, Goodwin LA (2013). Comparison of 26 sphingomonad genomes reveals diverse environmental adaptations and biodegradative capabilities. Appl Environ Microbiol.

[6] Gan HM, Hudson AO, Rahman AYA, Chan KG, Savka MA (2013). Comparative genomic analysis of six bacteria belonging to the genus *Novosphingobium*: insights into marine adaptation, cell-cell signaling and bioremediation. BMC Genomics.

[7] Oelschlagel M, Ruckert C, Kalinowski J, Schmidt G, Schlomann M, Tischler D. Description of *Sphingopyxis fribergensis* sp. nov. - a soil bacterium with the ability to degrade styrene and phenylacetic acid. Int J Syst Evol Microbiol. 2015;65:3008-15. 10.1099/ijs.0.000371 26040579

[8] Yoon JH, Lee CH, Yeo SH, Oh TK (2005). *Sphingopyxis baekryungensis* sp. nov., an orange-pigmented bacterium isolated from sea water of the Yellow Sea in Korea. Int J Syst Evol Microbiol.

[9] Gan HY, Gan M, Tarasco M, Busairi I, Barton HA, Hudson AO (2014). Whole-genome sequences of five oligotrophic bacteria isolated from deep within Lechuguilla Cave, New Mexico. Genome Announc.

[10] Kim J, Kim J, Kim H, Kim I, Moon Y, Park S (2014). Draft genome sequence of *Sphingopyxis* sp. Strain MWB1, a crude-Oil-degrading marine bacterium. Genome Announc.

[11] Williams TJ, Ertan H, Ting L, Cavicchioli R (2009). Carbon and nitrogen substrate utilization in the marine bacterium *Sphingopyxis alaskensis* strain RB2256. ISME J.

[12] Hernáez MJ, Reineke W, Santero E (1999). Genetic analysis of biodegradation of tetralin by a *Sphingomonas* strain. Appl Environ Microbiol.

[13] López-Sánchez A, Floriano B, Andújar E, Hernáez MJ, Santero E (2010). Tetralin-induced and ThnR-regulated aldehyde dehydrogenase and β-oxidation genes in *Sphingomonas macrogolitabida* strain TFA. Appl Environ Microbiol.

[14] López-Sánehez A, Rivas-Marín E, Martínez-Pérez O, Floriano B, Santero E (2009). Co-ordmated regulation of two divergent promoters through higher-order complex formation by the LysR-type regulator ThnR. Mol Microbiol.

[15] Gao F, Zhang C-T (2008). Ori-Finder: a web-based system for finding *oriC*s in unannotated bacterial genomes. BMC Bioinformatics.

[16] Gao F, Luo H, Zhang C-T (2013). DoriC 5.0: an updated database of *oriC* regions in both bacterial and archaeal genomes. Nucleic Acids Res.

[17] Ozaki S, Katayama T (2012). Highly organized DnaA-*oriC* complexes recruit the single-stranded DNA for replication initiation. Nucleic Acids Res.

[18] Brassinga AK, Siam R, Marczynski GT (2001). Conserved gene cluster at replication origins of the alpha-proteobacteria *Caulobacter crescentus* and *Rickettsia prowazekii*. J Bacteriol.

[19] Zhang Y, Romero H, Salinas G, Gladyshev VN (2006). Dynamic evolution of selenocysteine utilization in bacteria: a balance between selenoprotein loss and evolution of selenocysteine from redox active cysteine residues. Genome Biol.

[20] Hyatt D, Chen G-L, Locascio PF, Land ML, Larimer FW, Hauser LJ (2010). Prodigal: prokaryotic gene recognition and translation initiation site identification. BMC Bioinformatics.

[21] Muñoz-Mérida A, Viguera E, Claros MG, Trelles O, Pérez-Pulido AJ (2014). Sma3s: a three-step modular annotator for large sequence datasets. DNA Res.

[22] Altschul SF, Gish W, Miller W, Myers EW, Lipman DJ (1990). Basic local alignment search tool. J Mol Biol.

[23] Yabuuchi E (2002). Correction of the connecting vowel and gender *Sphingomonas macrogoltabidus* Takeuchi et al. 1993 to *Sphingomonas* macrogolitabida. Int J Syst Evol Microbiol.

[24] Konstantinidis KT, Tiedje JM (2005). Towards a genome-based taxonomy for prokaryotes. J Bacteriol.

[25] Vesth T, Lagesen K, Acar Ö, Ussery D (2013). CMG-biotools, a free workbench for basic comparative microbial genomics. PLoS One.

[26] Kim O-S, Cho Y-J, Lee K, Yoon S-H, Kim M, Na H (2012). Introducing EzTaxon-e: a prokaryotic 16S rRNA gene sequence database with phylotypes that represent uncultured species. Int J Syst Evol Microbiol.

[27] Funatsu G, Wittmann HG (1972). Ribosomal proteins. J Mol Biol.

[28] Finken M, Kirschner P, Meier A, Wrede A, Böttger EC (1993). Molecular basis of streptomycin resistance in *Mycobacterium tuberculosis*: alterations of the ribosomal protein S12 gene and point mutations within a functional 16S ribosomal RNA pseudoknot. Mol Microbiol.

[29] Alikhan N-F, Petty NK, Ben Zakour NL, Beatson SA (2011). BLAST Ring Image Generator (BRIG): simple prokaryote genome comparisons. BMC Genomics.

[30] Dhillon BK, Laird MR, Shay JA, Winsor GL, Lo R, Nizam F (2015). IslandViewer 3: more flexible, interactive genomic island discovery, visualization and analysis. Nucleic Acids Res.

[31] Stolz A (2014). Degradative plasmids from sphingomonads. FEMS Microbiol Lett.

[32] Bi D, Xu Z, Harrison EM, Tai C, Wei Y, He X (2011). ICEberg: a web-based resource for integrative and conjugative elements found in Bacteria. Nucleic Acids Res.

[33] Zhou Y, Liang Y, Lynch KH, Dennis JJ, Wishart DS (2011). PHAST: a fast phage search tool. Nucleic Acids Res.

[34] Grissa I, Vergnaud G, Pourcel C (2007). CRISPRFinder: a web tool to identify clustered regularly interspaced short palindromic repeats. Nucleic Acids Res.

[35] Caspi R, Altman T, Billington R, Dreher K, Foerster H, Fulcher CA (2014). The MetaCyc database of metabolic pathways and enzymes and the BioCyc collection of Pathway/Genome Databases. Nucleic Acids Res.

[36] Karp PD, Paley S, Romero P (2002). The pathway tools software. Bioinforma.

[37] Medema MH, Blin K, Cimermancic P, de Jager V, Zakrzewski P, Fischbach MA (2011). antiSMASH: rapid identification, annotation and analysis of secondary metabolite biosynthesis gene clusters in bacterial and fungal genome sequences. Nucleic Acids Res.

[38] Kim MK, Im W-T, Ohta H, Lee M, Lee S-T (2005). *Sphingopyxis granuli* sp. nov., a β-glucosidase-producing bacterium in the family *Sphingomonadaceae* in α-4 subclass of the Proteobacteria. J Microbiol.

[39] Crosson S, McGrath PT, Stephens C, McAdams HH, Shapiro L (2005). Conserved modular design of an oxygen sensory/signaling network with species-specific output. Proc Natl Acad Sci.

[40] Rowe JJ, Ubbink-Kok T, Molenaar D, Konings WN, Driessen AJM (1994). Nark is a nitrite-extrusion system involved in anaerobic nitrate respiration by *Escherichia coli*. Mol Microbiol.

[41] Patureau D, Zumstein E, Delgenes JP, Moletta R (2000). Aerobic denitrifiers isolated from diverse natural and managed ecosystems. Microb Ecol.

[42] Gish W, States DJ (1993). Identification of protein coding regions by database similarity search. Nat Genet.

[43] Yu Z, Li T, Zhao J, Luo J (2002). PGAAS: a prokaryotic genome assembly assistant system. Bioinforma.

[44] Wu S, Zhu Z, Fu L, Niu B, Li W (2011). WebMGA: a customizable web server for fast metagenomic sequence analysis. BMC Genomics.

[45] Laslett D, Canback B (2004). ARAGORN, a program to detect tRNA genes and tmRNA genes in nucleotide sequences. Nucleic Acids Res.

[46] Lowe TM, Eddy SR (1997). tRNAscan-SE: a program for improved detection of transfer RNA genes in genomic sequence. Nucleic Acids Res.

[47] Kinouchi M, Kurokawa K (2006). tRNAfinder: a software system to find all tRNA genes in the DNA sequence based on the cloverleaf secondary structure. J Comput Aided Chem.

[48] Tåquist H, Cui Y, Ardell DH (2007). TFAM 1.0: an online tRNA function classifier. Nucleic Acids Res.

[49] Lagesen K, Hallin P, Rodland EA, Staerfeldt H-H, Rognes T, Ussery DW (2007). RNAmmer: consistent and rapid annotation of ribosomal RNA genes. Nucleic Acids Res.

[50] Nawrocki EP, Eddy SR (2013). Infernal 1.1: 100-fold faster RNA homology searches. Bioinformatics.

[51] Richter M, Rosselló-Móra R (2009). Shifting the genomic gold standard for the prokaryotic species definition. Proc Natl Acad Sci U S A.

[52] Gouy M, Guindon S, Gascuel O (2010). SeaView Version 4: a multiplatform graphical user interface for sequence alignment and phylogenetic tree building. Mol Biol Evol.

[53] Waack S, Keller O, Asper R, Brodag T, Damm C, Fricke WF (2006). Score-based prediction of genomic islands in prokaryotic genomes using hidden Markov models. BMC Bioinformatics.

[54] Hsiao WWL, Ung K, Aeschliman D, Bryan J, Finlay BB, Brinkman FSL (2005). Evidence of a large novel gene pool associated with prokaryotic genomic islands. PLoS Genet.

[55] Dorn E, Hellwig M, Reineke W, Knackmuss HJ (1974). Isolation and characterization of a 3-chlorobenzoate degrading pseudomonad. Arch Microbiol.

[56] Tomás-Gallardo L, Canosa I, Santero E, Camafeita E, Calvo E, López JA (2006). Proteomic and transcriptional characterization of aromatic degradation pathways in *Rhodoccocus* sp. strain TFB. Proteomics.

[57] König C, Eulberg D, Gröning J, Lakner S, Seibert V, Kaschabek SR (2004). A linear megaplasmid, p1CP, carrying the genes for chlorocatechol catabolism of *Rhodococcus opacus* 1CP. Microbiology.

[58] Fischer M, Alderson J, van Keulen G, White J, Sawers RG (2010). The obligate aerobe *Streptomyces coelicolor* A3(2) synthesizes three active respiratory nitrate reductases. Microbiology.

