# Supplementary material for: Genomic analysis of the nitrate-respiring Sphingopyxis granuli (formerly Sphingomonas macrogoltabida) strain TFA
Source: BMC Genomics. 2016 Feb 4;17:93. doi: 10.1186/s12864-016-2411-1 (PMC4741004; doi:10.1186/s12864-016-2411-1)
Supplement: Additional file 1: — Analysis of the oriC genomic region. A. Genetic organization around the predicted oriC region in several Sphingomonadaceae representatives. The oriC region (line in purple) is predicted between yqfL (in black) and hemE (in red). Two other genes, encoding a membrane protein (in blue) and the Rho factor (in green), are also conserved. Non-conserved genes are in gray. B. Clustal alignment of the predicted oriC sequences of Sphingopyxis strains with sequenced genomes. Recognized DnaA binding boxes, predicted by OriC Finder, are indicated in gray and the putative duplex unwinding element (DUE) is highlighted in black. (PDF 70 kb) [file 12864_2016_2411_MOESM1_ESM.pdf]

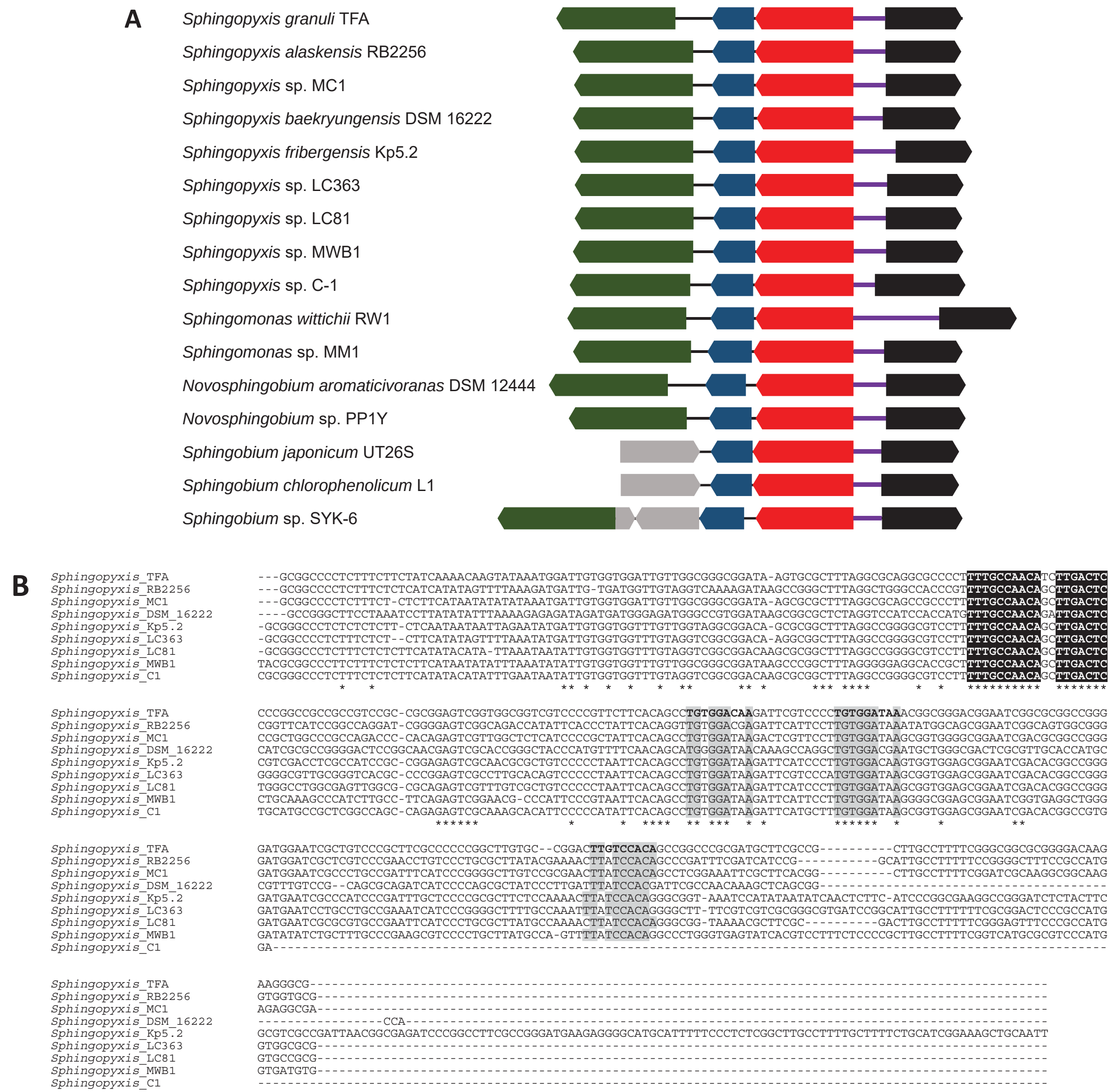
A
Sphingopyxis granuli TFA
Sphingopyxis alaskensis RB2256
Sphingopyxis sp. MC1
Sphingopyxis baekryungensis DSM 16222
Sphingopyxis fribergensis Kp5.2
Sphingopyxis sp. LC363
Sphingopyxis sp. LC81
Sphingopyxis sp. MWB1
Sphingopyxis sp. C-1
Sphingomonas wittichii RW1
Sphingomonas sp. MM1
Novosphingobium aromaticivoranas DSM 12444
Novosphingobium sp. PP1Y
Sphingobium japonicum UT26S
Sphingobium chlorophenolicum L1
Sphingobium sp. SYK-6
B
Sphingopyxis_TFA ---GCGGCCCCTCTTTCTTCTATCAAAACAAGTATAAATGGATTGTGGTGGATTGTTGGCGGGCGGATA-AGTGCGCTTTAGGCGCAGGCGCCCCTTTTGCCAACATCTTGACTC
Sphingopyxis_RB2256 ---GCGGCCCCTCTTTCTCTCATCATATAGTTTTAAAGATGATTG-TGATGGTTGTAGGTCAAAAGATAAGCCGGGCTTTAGGCTGGGCCACCCGTTTTGCCAACAGCTTGACTC
Sphingopyxis_MC1 ---GCGGCCCCTCTTTCT-CTCTTCATAATATATATAAATGATTGTGGTGGATTGTTGGCGGGCGGATA-AGCGCGCTTTAGGCGCAGCCGCCCTTTTTGCCAACAGCTTGACTC
Sphingopyxis_DSM_16222 ----GCCGGGCTTCCTAAATCCTTATATATTTAAAAGAGAGATAGATGATGGGAGATGGGCCGTGGATAAGCGGCGCTCTAGGTCCATCCACCATGTTTGCCAACAGCTTGACTC
Sphingopyxis_Kp5.2 -GCGGGCCCTCTCTCTCTT-CTCAATAATAATTAGAATATGATTGTGGTGGTTTGTTGGTAGGCGGACA-GCGCGGCTTTAGGCCGGGGCGTCCTTTTTGCCAACAGCTTGACTC
Sphingopyxis_LC363 -GCGGCCCCTCTTTCTCT--CTTCATATAGTTTAAATATGATTGTGGTGGTTTGTAGGTCGGCGGACA-AGGCGGCTTTAGGCCGGGGCGTCCTTTTTGCCAACAGCTTGACTC
Sphingopyxis_LC81 -GCGGGCCCTCTTTCTCTCTTCATATACATA-TTAAATAATATTGTGGTGGTTTGTAGGTCGGCGGACAAGCGCGGCTTTAGGCCGGGGCGTCCTTTTTGCCAACAGCTTGACTC
Sphingopyxis_MWB1 TACGCGGCCCTTCTTTCTCTCTTCATAATATATTTAAATATATTGTGGTGGTTTGTTGGCGGGCGGATAAGCCCGGCTTTAGGGGGAGGCACCGCTTTTGCCAACAGCTTGACTC
Sphingopyxis_C1 CGCGGGCCCTCTTTCTCTCTTCATATACATATTTGAATAATATTGTGGTGGTTTGTAGGTCGGCGGACAAGCGCGGCTTTAGGCCGGGGCGTCCTTTTTGCCAACAGCTTGACTC
Sphingopyxis_TFA CCCGGCCGCCGCCGTCCGC-CGCGGAGTCGGTGGCGGTCGTCCCCGTTCTTCACAGCCTGTGGACAAGATTCGTCCCCTGTGGATAAACGGCGGGACGGAATCGGCGCGGCCGGG
Sphingopyxis_RB2256 CGGTTCATCCGGCCAGGAT-CGGGGAGTCGGCAGACCATATTCACCCTATTCACAGGTTGTGGACGAGATTCATTCCTTGTGGATAAATATGGCAGCGGAATCGGCAGTGGCGGG
Sphingopyxis_MC1 CCGCTGGCCCGCCAGACCC-CACAGAGTCGTTGGCTCTCATCCCCGCTATTCACAGCCTGTGGATAAGACTCGTTCCTTGTGGATAAGCGGTGGGGCGGAATCGACGCGGCCGGG
Sphingopyxis_DSM_16222 CATCGCGCGGGGACTCCGGCAACGAGTCGCACCGGGCTACCCATGTTTTCAACAGCATGGGGATAACAAAGCCAGGCTGTGGACGAATGCTGGGCGACTCGCGTTGCACCATGC
Sphingopyxis_Kp5.2 CGTCGACCTCGCCATCCGC-CGGAGAGTCGCAACGCGCTGTCCCCCTAATTCACAGCCTGTGGATAAGATTCATCCCTTGTGGACAAGTGGTGGAGCGGAATCGACACGGCCGGG
Sphingopyxis_LC363 GGGGCGTTGCGGGTCACGC-CCCGGAGTCGCCTTGCACAGTCCCCCTAATTCACAGCCTGTGGATAAGATTCGTCCCATGTGGATAAGCGGTGGAGCGGAATCGACACGGCCGGG
Sphingopyxis_LC81 TGGGCCTGGCGAGTTGGCG-CGCAGAGTCGTTTGTCGCTGTCCCCCTAATTCACAGCCTGTGGATAAGATTCATCCCTTGTGGATAAGCGGTGGAGCGGAATCGACACGGCCGGG
Sphingopyxis_MWB1 CTGCAAAGCCCATCTTGCC-TTCAGAGTCGGAACG-CCCATTCCCCGTAATTCACAGCCTGTGGATAAGATTCATTCCTTGTGGATAAGGGGCGGAGCGGAATCGGTGAGGCTGGG
Sphingopyxis_C1 TGCATGCCGCTCGGCCAGC-CAGAGAGTCGCAAAGCACATTCCCCCATATTCACAGCCTGTGGATAAGATTCATGCTTGTGGATAAGCGGTGGAGCGGAATCGACACGGCCGTG
Sphingopyxis_TFA GATGGAATCGCTGTCCCGCTTCGCCCCCCGGCTTGTGC--CGGACTTGTCCACAGCCGGCCCGCGATGCTTCGCCG----------CTTGCCTTTTCGGGCGGCGCGGGGACAAG
Sphingopyxis_RB2256 GATGGATCGCTCGTCCCGAACCTGTCCCTGCGCTTATACGAAAACTTATCCACAGCCCGATTTCGATCATCCG-----------GCATTGCCTTTTTCCGGGGCTTTCCGCCATG
Sphingopyxis_MC1 GATGGAATCGCCCTGCCGATTTCATCCCGGGGCTTGTCCGCGAACTTATCCACAGCCTCGGAAATTCGCTTCACGG----------CTTGCCTTTTCGGATCGCAAGGCGGCAAG
Sphingopyxis_DSM_16222 CGTTGTCCG--CAGCGCAGATCATCCCAGCGCTATCCCTTGATTTATCCACGATTCGCCAACAAAGCTCAGCGG--
Sphingopyxis_Kp5.2 GATGAATCGCCCATCCCGATTTGCTCCCCGCGCTTCTCCAAAACTTATCCACAGGGCGGT-AAATCCATATAATATCAACTCTTC-ATCCCGGCGAAGGCCGGGATCTCTACTTC
Sphingopyxis_LC363 GATGAATCCTGCCTGCCGAAATCATCCCGGGGCTTTTGCCAAATTTATCCACAGGGGCTT-TTCGTCGTCGCGGGCGTGATCCGGCATTGCCTTTTTTCGCGGACTCCCGCCATG
Sphingopyxis_LC81 GATGAATCGCGCGTGCCGAATTCATCCCTGCGCTTATGCCAAAACTTATCCACAGGGCGG-TAAAACGCTTCGC----------GACTTGCCTTTTTCGGGAGTTTCCCGCCATG
Sphingopyxis_MWB1 GATATATCTGCTTTGCCCGAAGCGTCCCCTGCTTATGCCA-GTTTTATCCACAGGCCCTGGGTGAGTATCACGTCCTTTCTCCCCGCTTGCCTTTTCGGTCATGCGCGTCCCATG
Sphingopyxis_C1 GA---
Sphingopyxis_TFA AAGGGCG--
Sphingopyxis_RB2256 GTGGTGCG---
Sphingopyxis_MC1 AGAGGCGA---
Sphingopyxis_DSM_16222 -----------------CCA---
Sphingopyxis_Kp5.2 GCGTCGCCGATTAACGGCGAGATCCCGGCCTTCGCCGGGATGAAGAGGGGCATGCATTTTTCCCTCTCGGCTTGCCTTTTGCTTTTCTGCATCGGAAAGCTGCAATT
Sphingopyxis_LC363 GTGGCGCG---
Sphingopyxis_LC81 GTGCCGCG---
Sphingopyxis_MWB1 GTGATGTG---
Sphingopyxis_C1 ---
