# Supplementary figures and images for: Genomic analysis of the nitrate-respiring Sphingopyxis granuli (formerly Sphingomonas macrogoltabida) strain TFA
Source: BMC Genomics. 2016 Feb 4;17:93. doi: 10.1186/s12864-016-2411-1 (PMC4741004; doi:10.1186/s12864-016-2411-1)

**COG Classification**  
*Sphingopyxis* core genome

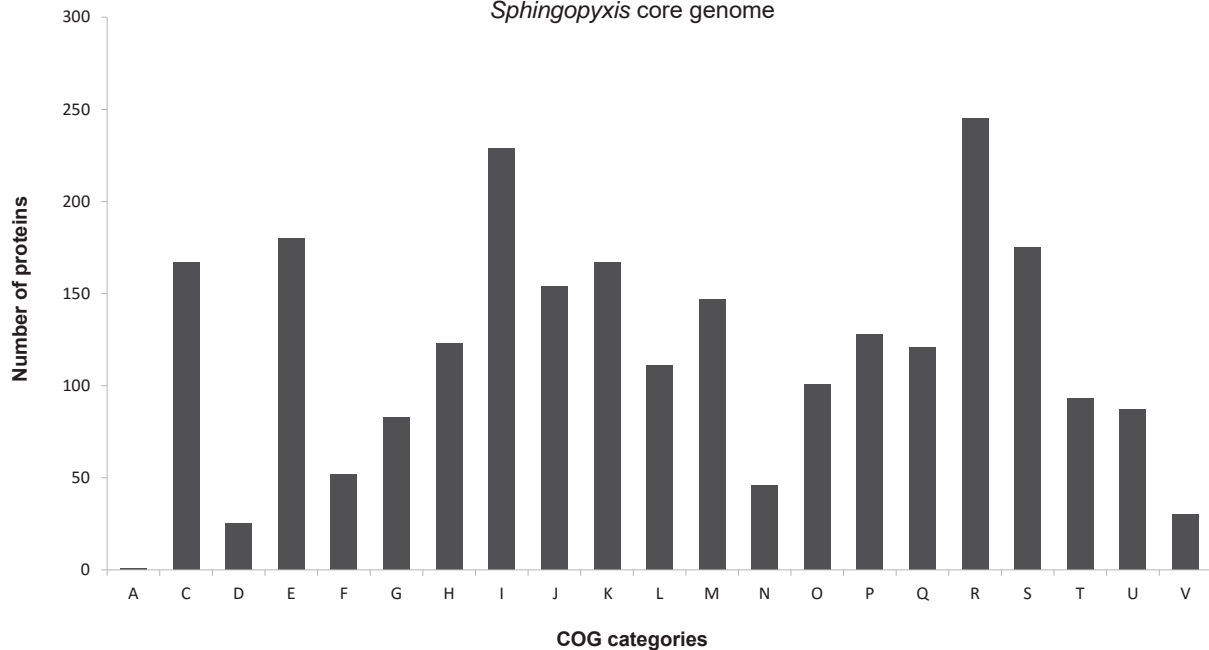

Supplement: Additional file 2: — Classification proteins encoded by the Sphingopyxis genus core genome in COG categories. COG categories are A, RNA processing and modification; C, Energy production and conversion; D, Cell cycle control, cell division, chromosome partitioning; E, Amino acid transport and metabolism; F, Nucleotide transport and metabolism; G, Carbohydrate transport and metabolism; H, Coenzyme transport and metabolism; I, Lipid transport and metabolism; J, Translation, ribosomal structure and biogenesis; K, Transcription; L, Replication, recombination and repair; M, Cell wall/membrane/envelope biogenesis; N, Cell motility; O, Posttranslational modification, protein turnover, chaperones; P, Inorganic ion transport and metabolism; Q, Secondary metabolites biosynthesis, transport and catabolism; R, General function prediction only; S, Function unknown; T, Signal transduction mechanisms; U, Intracellular trafficking, secretion, and vesicular transport; V, Defense mechanisms. (PDF 63 kb) [file 12864_2016_2411_MOESM2_ESM.pdf]

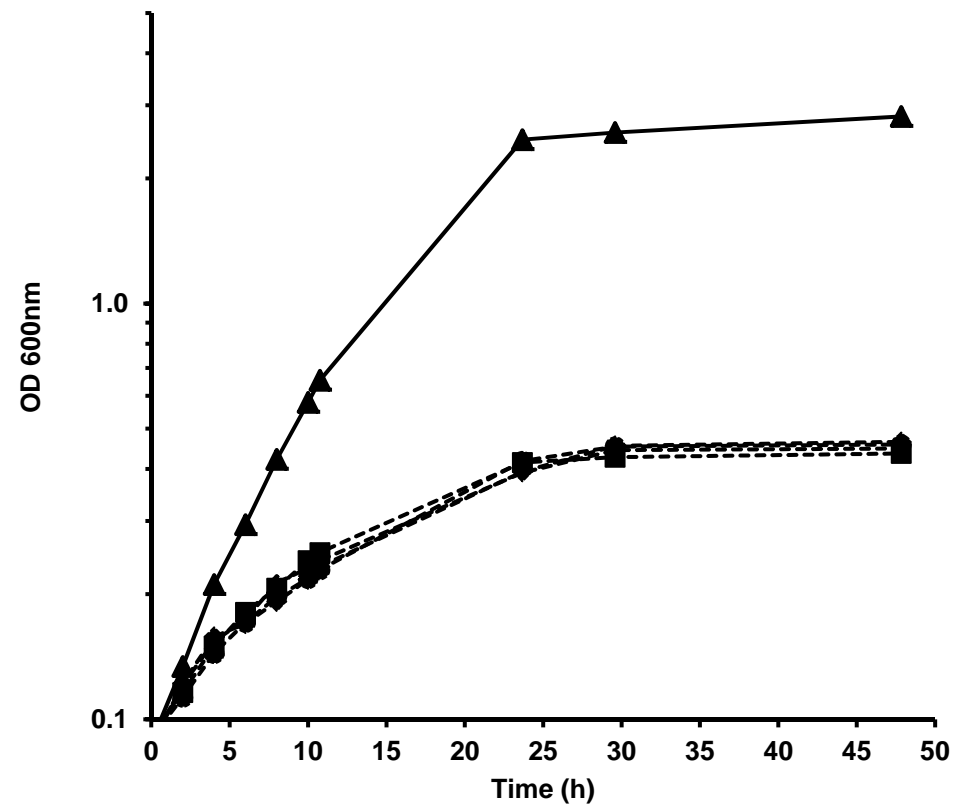

Supplement: Additional file 7: — Aerobic growth of TFA using different nitrogen sources: ammonium 15 mM (triangles), nitrate 20 mM (squares), nitrite 10 mM (circles), nitrite 5 mM (diamonds) and without nitrogen source added (cross). (PDF 28 kb) [file 12864_2016_2411_MOESM7_ESM.pdf]
