# Supplementary material for: Genomic analysis of the nitrate-respiring Sphingopyxis granuli (formerly Sphingomonas macrogoltabida) strain TFA
Source: BMC Genomics. 2016 Feb 4;17:93. doi: 10.1186/s12864-016-2411-1 (PMC4741004; doi:10.1186/s12864-016-2411-1)
Supplement: Additional file 3: — Accession numbers of bacterial genomes used in this paper. (DOCX 16 kb) [file 12864_2016_2411_MOESM3_ESM.docx]

Accession Numbers of the genomes used in this study

| Genome | Completeness | No. of contigs (plasmids excluded) | NCBI accession numbers |
| --- | --- | --- | --- |
| *Sphingopyxis* *granuli* TFA | Complete | 1 | CP012199 (This Study) |
| *Sphingopyxis* sp. MC1 | Draft | 24 | NZ_AOUN01000001-NZ_AOUN01000024 |
| *Sphingopyxis alaskensis* RB2256 | Complete | 1 | NC_008048.1 |
| *Sphingopyxis fribergensis* Kp5.2 | Complete | 1 | NZ_CP009122.1 |
| *Sphingopyxis* sp. LC363 | Draft | 73 | NZ_JNFC01000001-NZ_JNFC01000073 |
| *Sphingopyxis* sp. LC81 | Draft | 48 | NZ_JNFD01000001-NZ_JNFD01000048 |
| *Sphingopyxis* sp. MWB1 | Draft | 5 | NZ_JQFJ01000001-NZ_JQFJ01000005 |
| Sphingopyxis sp. C1 | Draft | 2 | BBRO01000001-BBRO01000002 |
| *Sphingopyxis baekryungensis* DSM 16222 | Draft | 10 | NZ_ATUR01000001-NZ_ATUR01000010 |
| *Sphingomonas wittichii* RW1 | Complete | 1 | NC_009511.1 |
| *Novosphingobium aromaticivorans* DSM 12444 | Complete | 1 | NC_007794.1 |
| *Novosphingobium* sp. PP1Y | Complete | 1 | NC_015580.1 |
| *Erythrobacter litoralis* HTCC2594 | Complete | 1 | NC_007722.1 |
| *Blastomonas* sp. AAP53 | Draft | 28 | NZ_ANFZ01000001-NZ_ANFZ01000028 |
| *Sphingobium* sp. SYK-6 | Complete | 1 | NC_015976.1 |
| *Sphingobium japonicum* UT26S | Compete | 2 | NC_014006.1, NC_014013.1 |
| *Sphingobium chlorophenolicum* L-1 | Complete | 2 | NC_015593.1, NC_015594.1 |
| *Oligotropha carboxidovorans* OM4 | Complete | 1 | NC_017538.1 |
| *Oligotropha carboxidovorans* OM5 | Complete | 1 | NC_015684.1 |
| *Zymomonas mobilis subsp. mobilis* ATCC 10988 | Complete | 1 | NC_017262.1 |
| *Sphingomonas sanxanigenens* NX02 | Complete | 1 | NZ_CP006644.1 |
| *Sphingomonas sp.* MM-1 | Complete | 1 | NC_020561.1 |
