# Supplementary material for: Genomic analysis of the nitrate-respiring Sphingopyxis granuli (formerly Sphingomonas macrogoltabida) strain TFA
Source: BMC Genomics. 2016 Feb 4;17:93. doi: 10.1186/s12864-016-2411-1 (PMC4741004; doi:10.1186/s12864-016-2411-1)
Supplement: Additional file 4: — Genes present in TFA genomic regions highly similar to other α-proteobacteria genomes. (PDF 83 kb) [file 12864_2016_2411_MOESM4_ESM.pdf]

	S. granuli TFA						*S. alaskensis* RBRB2256			
	**Locus_tag**	**start**	**stop**	**strand**	**gene_name**	**Description**	**Alignment_position**	**Strand**	**gene_name**	**Locus_tag**
	SGRAN_0122	134249	133773	-		DNA-binding domain-containing protein, AraC-type	25506..25982	+	-	Sala_2430
	SGRAN_0123	136614	134293	-	*copA1*	Heavy metal translocating P-type ATPase	23132..25453	+	-	Sala_2429
	SGRAN_0124	137458	136781	-		Methyltransferase type 12	22288..22965	+	-	Sala_2428
	SGRAN_0125	138285	137596	-		Transcriptional regulator, AraC family	21461..22150	+	-	Sala_2427
	SGRAN_0126	139286	138318	-	*copD2*	Copper resistance D	20460..21428	+	-	Sala_2426
	SGRAN_0127	139676	139293	-		Copper resistance protein CopC	20070..20453	+	-	Sala_2425
	SGRAN_0128	139820	140086	+		Uncharacterized protein	19220..19657	-	-	Sala_2424
	SGRAN_0129	140089	140526	+		Nickel-cobalt-cadmium resistance protein nccX				
	SGRAN_0130	140523	141107	+		ECF subfamily RNA polymerase sigma-24 factor	18639..19223	-	-	Sala_2423
	SGRAN_0131	141281	143062	+	*copA2*	Copper-resistance protein, CopA family	16684..18465	-	-	Sala_2422
	SGRAN_0132	143059	144270	+	*pcoB*	Copper resistance B	15476..16687	-	-	Sala_2421
**Similarity 1**	SGRAN_0133	144309	144752	+		Uncharacterized protein	14994..15437	-	-	Sala_2420
	SGRAN_0134	144754	145278	+	*copG*	CopG protein				
	SGRAN_0135	145305	145652	+		Uncharacterized protein	14468..14992	-	-	Sala_2419
	SGRAN_0136	145624	145779	+		Uncharacterized protein	13965..14423	+	-	Sala_2418
	SGRAN_0137	145896	148367	+		TonB-dependent receptor, plug	11381..13852	-	-	Sala_2417
	SGRAN_0138	149309	148404	-	*lysR*	Transcriptional regulator, LysR family	10439..11344	+	-	Sala_2416
	SGRAN_0139	149365	150435	+	*aapJ*	Glutamate Aspartate periplasmic binding protein GltI	9313..10410	-	-	Sala_2415
	SGRAN_0140	150432	152624	+	*aapM*	Amino acid ABC transporter permease	7124..9316	-	-	Sala_2414
	SGRAN_0141	152614	153861	+	*metC2*	Cystathionine beta-lyase	5887..7134	-	-	Sala_2413
	SGRAN_0142	153858	154610	+	*metN*	ABC-type polar amino acid transport system, ATPase component	5138..5890	-	-	Sala_2412
	SGRAN_0143	154897	157599	+		TonB-dependent receptor	2147..4852	-	-	Sala_2411
	SGRAN_0144	157611	158315	+		Uncharacterized conserved secreted/membrane protein	1431..2135	-	-	Sala_2410
	SGRAN_0145	158312	159046	+		PepSY-associated TM helix	700..1434	-	-	Sala_2409
							1..693	+	-	Sala_2408

	S. granuli TFA						*Oligothropha caboxidovorans* OM4			
	**Locus_tag**	**start**	**stop**	**strand**	**gene_name**	**Description**	**Alignment_position**	**Strand**	**gene_name**	**Locus_tag**
	SGRAN_0160	173796	173990	+		Uncharacterized protein	46026..46220	-	-	OCA5_c03410
	SGRAN_0161	174045	174290	+		Uncharacterized protein	45724..45972	-	-	OCA5_c03400
	SGRAN_0162	174896	176599	+	*kdpA*	Potassium-transporting ATPase A chain	43421..45118	-	kdpA	OCA5_c03390
	SGRAN_0163	176608	176784	+		Uncharacterized protein				
	SGRAN_0164	176827	178896	+	*kdpB*	Potassium-transporting ATPase B chain	41124..43193	-	kdpB	OCA5_c03380
	SGRAN_0165	178969	179541	+	*kdpC*	Potassium-transporting ATPase C chain	40473..41048	-	kdpC	OCA5_c03370
	SGRAN_0166	179600	182305	+	*kdpD*	Sensor protein KdpD	37711..40416	-	kdpD	OCA5_c03360
	SGRAN_0167	182302	182997	+	*kdpE*	Transcriptional regulatory protein KdpE	37019..37714	-	kdpE	OCA5_c03350
	SGRAN_0168	183045	183497	+	*ptsN2*	Nitrogen regulatory protein PtsN	36519..36971	-	ptsN2	OCA5_c03340
	SGRAN_0169	183494	183883	+	*ibpA*	Putative small heat shock protein	36076..36522	-	-	OCA5_c03330
	SGRAN_0170	184317	185108	+		Transcriptional regulator, LysR family	34908..35891	-	-	OCA5_c03320
	SGRAN_0171	185280	185041	-		Uncharacterized protein	34736..34975	+	-	OCA5_c03310
	SGRAN_0172	186466	185282	-	*trbI1*	Conjugal transfer protein TrbI	33550..34734	+	trbI1	OCA5_c03300
	SGRAN_0173	187446	186463	-	*trbG1*	Conjugal transfer protein TrbG	32570..33553	+	trbG1	OCA5_c03290
	SGRAN_0174	188132	187443	-	*trbF1*	Conjugal transfer protein TrbF	31884..32573	+	trbF1	OCA5_c03280
	SGRAN_0175	189493	188129	-	*trbL1*	Conjugal transfer protein TrbL	30523..31887	+	trbL1	OCA5_c03270
	SGRAN_0176	189781	189497	-		Conjugal transfer protein TrbK	30235..30519	+	-	OCA5_c03260
	SGRAN_0177	190558	189791	-	*trbJ1*	P-type conjugal transfer protein TrbJ	29457..30224	+	trbJ1	OCA5_c03250
	SGRAN_0178	193014	190561	-	*trbE1*	Conjugal transfer protein TrbE	27001..29454	+	trbE1	OCA5_c03240
	SGRAN_0179	193309	193028	-	*trbD1*	Conjugal transfer protein TrbB	26374..26706	+	trbC	OCA5_c03230
	SGRAN_0180	193641	193309	-	*trbC1*	Putative conjugal transfer protein TrbC	25397..26377	+	trbB1	OCA5_c03220
	SGRAN_0181	194618	193638	-	*trbB1*	P-type conjugative transfer ATPase TrbB	25105..25344	+	-	OCA5_c03210

	SGRAN_0182	195339	194914	-		Helix-turn-helix protein, CopG	24676..25101	+	-	OCA5_c03200
**Similarity 2**	SGRAN_0183	197329	195344	-	*traG1*	Conjugal transfer protein traG	22686..24671	+	traG1	OCA5_c03190
	SGRAN_0184	199217	197478	-	*virD22*	Uncharacterized protein	20798..22537	+	-	OCA5_c03180
	SGRAN_0185	200183	199461	-	*slt2*	Lytic transglycosylase catalytic	19832..20554	+	-	OCA5_c03170
	SGRAN_0186	200520	200188	-		Uncharacterized protein	19495..19827	+	-	OCA5_c03160
	SGRAN_0187	201105	200560	-	*traF1*	Putative conjugal transfer protein TraF	18910..19455	+	traF1	OCA5_c03150
	SGRAN_0188	201623	201102	-		Uncharacterized protein	18392..18913	+	-	OCA5_c03140
	SGRAN_0189	201871	201620	-	*parG*	Plasmid segregation centromere-binding protein ParG	18144..18395	+	-	OCA5_c03130
	SGRAN_0190	202521	201868	-	*parA*	Cobyrinic acid ac-diamide synthase	17494..18147	+	-	OCA5_c03120
	SGRAN_0191	203582	202518	-	*repA*	Replication protein A	16337..17497	+	-	OCA5_c03110
	SGRAN_0192	203977	203696	-		Putative transcriptional regulator	16038..16319	+	-	OCA5_c03100
	SGRAN_0193	204475	204107	-		Uncharacterized protein	15201..15908	+	-	OCA5_c03090
	SGRAN_0194	205266	205006	-		Uncharacterized protein	14749..15009	+	-	OCA5_c03080
	SGRAN_0195	206143	205817	-		Uncharacterized protein	14390..14638	+	-	OCA5_c03070
	SGRAN_0196	207150	206710	-		Uncharacterized protein	13836..14198	+	-	OCA5_c03060
	SGRAN_0197	207454	207287	-		Uncharacterized protein	12865..13305	+	-	OCA5_c03050
	SGRAN_0198	207662	207979	+		Uncharacterized protein	12561..12728	+	-	OCA5_c03040
	SGRAN_0199	209169	208243	-		Uncharacterized protein	10851..11777	+	-	OCA5_c03030
	SGRAN_0200	210558	209515	-		Uncharacterized protein	9462..10505	+	-	OCA5_c03020
	SGRAN_0201	214886	210555	-		Putative methylase/helicase	5134..9465	+	-	OCA5_c03010
	SGRAN_0202	215688	215044	-		Uncharacterized protein	4332..4976	+	-	OCA5_c03000
	SGRAN_0203	216097	215681	-		Uncharacterized protein	3923..4339	+	-	OCA5_c02990
	SGRAN_0204	216718	216161	-		Uncharacterized protein	3302..3859	+	-	OCA5_c02980
	SGRAN_0205	217048	216827	-		Uncharacterized protein	2972..3193	+	-	OCA5_c02970
	SGRAN_0206	219240	217105	-		ParB domain protein nuclease	780..2915	+	-	OCA5_c02960
	SGRAN_0207	219789	219418	-		Uncharacterized protein	231..602	+	-	OCA5_c02950
	SGRAN_0208	220082	219786	-	*ardC*	Antirestriction protein	1..234	+	-	OCA5_c02940
	***S. granuli* TFA**						***Sphingobium chlorophenolicum* L1**			
	**Locus_tag**	**start**	**stop**	**strand**	**gene_name**	**Description**	**Alignment_position**	**Strand**	**gene_name**	**Locus_tag**
	SGRAN_0556	590566	591651	+		Putative uncharacterized protein	37095..38180	-		Sphch_0919
	SGRAN_0557	591788	591988	+		Putative uncharacterized protein	36758..36958	-		Sphch_0918
	SGRAN_0558	592080	593084	+		Uncharacterized protein	35662..36666	-		Sphch_0917
	SGRAN_0559	593372	593196	-		Putative uncharacterized protein	35374..35550	+		Sphch_0916
	SGRAN_0560	593607	594209	+		Uncharacterized protein	34537..35139	-		Sphch_0915
	SGRAN_0561	594297	594611	+		Prevent-host-death family protein	34135..34449	-		Sphch_0914
	SGRAN_0562	594608	595060	+	*vapC*	Probable ribonuclease VapC	33686..34138	-		Sphch_0913
							33511..33660	+		Sphch_0912
	SGRAN_0563	595921	595526	-		Putative uncharacterized protein	32822..33217	+		Sphch_0911
	SGRAN_0564	596020	596256	+		Uncharacterized protein	32471..32776	+		Sphch_0910
	SGRAN_0565	596831	597304	+		Putative uncharacterized protein	31437..31910	-		Sphch_0909
	SGRAN_0566	597388	598413	+		Putative uncharacterized protein	30328..31353	-		Sphch_0908
	SGRAN_0567	598901	598578	-		Putative uncharacterized protein	29840..30163	+		Sphch_0907
	SGRAN_0568	599366	599665	+		CopG-like domain-containing protein DNA-binding protein	29076..29375	-		Sphch_0906
	SGRAN_0569	599838	601988	+		Type IV secretion-system, TraD, DNA-binding domain protein	26753..28903	-		Sphch_0905
**Similarity 3**	SGRAN_0570	601985	604978	+	*trwC*	Conjugative relaxase domain protein	23763..26756	-		Sphch_0904
	SGRAN_0571	604988	605578	+		Restriction endonuclease	23163..23753	-		Sphch_0903
	SGRAN_0572	605593	606132	+		Lytic transglycosylase catalytic	22609..23148	-		Sphch_0902
	SGRAN_0573	606698	609079	+	*ppsA*	PEP synthase	19662..22043	-		Sphch_0901
	SGRAN_0574	609376	610389	+		UspA domain-containing protein	18352..19365	-		Sphch_0900
	SGRAN_0575	610393	618804	+		Carbohydrate binding protein	9937..18348	-		Sphch_0899
	SGRAN_0576	618916	619152	+		Uncharacterized protein	9589..9825	-		Sphch_0898

							9208..9534	-		Sphch_0897
	SGRAN_0577	621066	619606	-		Aminoglycoside phosphotransferase	7579..9135	+		Sphch_0896
	SGRAN_0578	621661	621167	-	*hspC2*	Heat shock protein Hsp20	7080..7574	+		Sphch_0895
	SGRAN_0579	622201	624024	+	*fixL*	PAS/PAC sensor signal transduction histidine kinase	4716..6539	-		Sphch_0894
	SGRAN_0580	624014	624628	+	*fixJ*	Two component transcriptional regulator, LuxR family	4112..4726	-		Sphch_0893
	SGRAN_0581	624691	625122	+		Response regulator receiver protein	3618..4049	-		Sphch_0892
	SGRAN_0582	625170	625580	+		Globin	3160..3570	-		Sphch_0891
	SGRAN_0583	625612	626619	+	*adhA*	Zinc-binding alcohol dehydrogenase family protein	2121..3128	-		Sphch_0890
	SGRAN_0584	626735	627568	+		UspA domain-containing protein	1172..2005	-		Sphch_0889
	SGRAN_0585	627605	628642	+	*adh*	L-iditol 2-dehydrogenase	98..1135	-		Sphch_0888
**Similarity 4**	**Locus_tag**	**start**	**stop**	**strand**	**gene_name**	**Description**	**Alignment_position**	**Strand**	**gene_name**	**Locus_tag**
	SGRAN_0659	707273	708064	+		Autoinducer-binding domain protein	13414..14205	-		Sphch_0886
	SGRAN_0660	708258	709043	+		Protein of unknwon function (DUF2893)	12435..13229	-		Sphch_0885
	SGRAN_0661	709048	709968	+	ync	Ync	11510..12430	-		Sphch_0884
	SGRAN_0662	710535	710182	-		Putative uncharacterized protein	10943..11296	+		Sphch_0883
	SGRAN_0663	711133	711582	+		Uncharacterized protein	9896..10345	-		Sphch_0882
	SGRAN_0664	715349	711579	-	traG2	DNA transfer and F pilus assembly protein TraG	6129..9899	+		Sphch_0881
	SGRAN_0665	716788	715364	-	traH	TraH family protein	4690..6114	+		Sphch_0880
	SGRAN_0666	717418	716834	-		Putative uncharacterized protein	4060..4644	+		Sphch_0879
	SGRAN_0667	718287	717415	-		Thioredoxin-related protein	3191..4063	+		Sphch_0878
	SGRAN_0668	718895	718284	-	traF2	Type IV secretory pathway protease TraF-like protein	2583..3194	+		Sphch_0877
	SGRAN_0669	719748	718867	-	trbC2	Type-F conjugative transfer system pilin assembly protein TrbC	1730..2611	+		Sphch_0876
	SGRAN_0670	720838	719720	-	traN2	Mating pair stabilization protein TraN	640..1758	+		Sphch_0875
	SGRAN_0671	722772	720835	-		Putative exported protein	1..643	+		Sphch_0874
**Similarity 5**	***S. granuli* TFA**						***Erythrobacter litoralis* HTCC2594**			
	**Locus_tag**	**start**	**stop**	**strand**	**gene_name**	**Description**	**Alignment_position**	**Strand**	**gene_name**	**Locus_tag**
	SGRAN_1593	1731543	1728811	-		TonB-dependent receptor	181..2865	-		ELI_14925
	SGRAN_1594	1732780	1731710	-		Acyl-CoA dehydrogenase family protein	3081..4151	-		ELI_14930
	SGRAN_1595	1734010	1732799	-		Acyl-CoA dehydrogenase family protein	4170..5381	-		ELI_14935
	SGRAN_1596	1734405	1734079	-	*fdxB*	Ferredoxin	5450..5776	-		ELI_14940
	SGRAN_1597	1735696	1734434	-	*p450*	Cytochrome P450	5805..7067	-		ELI_14945
	SGRAN_1598	1736009	1736626	+		Regulatory protein GntR, HTH	7269..7997	+		ELI_14950
	SGRAN_1599	1736677	1737819	+		Oxidoreductase, 2-nitropropane dioxygenase family protein	8075..9190	+		ELI_14955
	SGRAN_1600	1737838	1738593	+		Putative Uncharacterized protein involved in ubiquinonebiosynthesis	9272..9964	+		ELI_14960
	SGRAN_1601	1738590	1739762	+		Putative nonspecific lipid-transfer protein	9961..11133	+		ELI_14965
	SGRAN_1602	1739759	1740235	+		Putative nucleic-acid-binding protein containing a Zn-ribbon	11130..11606	+		ELI_14970
	SGRAN_1603	1740242	1741492	+		Putative acyl-CoA dehydrogenase family protein	11613..12863	+		ELI_14975
	SGRAN_1604	1741492	1742583	+		Putative acyl-CoA dehydrogenase	12863..13954	+		ELI_14980
	SGRAN_1605	1742647	1744257	+	*fadD19*	Long-chain-fatty-acid--CoA ligase	14018..15628	+		ELI_14985
	SGRAN_1606	1744254	1745891	+	*pamO3*	Cyclohexanone monooxygenase	15625..17262	+		ELI_14990
	SGRAN_1607	1745888	1746859	+		Hydrolase, putative	17259..18230	+		ELI_14995
	SGRAN_1608	1747279	1750215	+		TonB-dependent receptor	18665..21586	+		ELI_15000
	SGRAN_1609	1752173	1750656	-	*oprN2*	RND efflux system, outer membrane lipoprotein, NodT family	22027..23514	-		ELI_15005
	SGRAN_1610	1753734	1752181	-	*rmrB*	Drug resistance transporter, EmrB/QacA subfamily	23552..25105	-		ELI_15010
	SGRAN_1611	1754865	1753738	-	*rmrA*	Multidrug resistance efflux pump	25109..26257	-		ELI_15015
							26345..26524	+		ELI_15020
	SGRAN_1612	1755493	1756443	+	ED21_31999	ABC-type transport system periplasmic component	26860..27705	+		ELI_15025
	***S. granuli* TFA**						***Novosphingoboum* sp. strain PP1Y**			
	**Locus_tag**	**start**	**stop**	**strand**	**gene_name**	**Description**	**Alignment_position**	**Strand**	**gene_name**	**Locus_tag**
	SGRAN_2835	3076973	3076722	-			18486..18947	+		PP1Y_AT15325
	SGRAN_2836	3077484	3077023	-			17793..18137	-		PP1Y_AT15318

	SGRAN_2837	3077770	3078177	+			27n			
	SGRAN_2838	3078204	3078491	+	*traL2*		17479..17766	-		PP1Y_AT15312
	SGRAN_2839	3078504	3079076	+	*traE2*		16894..17466	-		PP1Y_AT15306
	SGRAN_2840	3079076	3079834	+	*traK2*		16136..16894	-		PP1Y_AT15299
	SGRAN_2841	3079827	3081161	+	*traB2*		14809..16143	-		PP1Y_AT15288
	SGRAN_2842	3081158	3082048	+	*dsbC*		13922..14812	-		PP1Y_AT15277
	SGRAN_2843	3082054	3082797	+	*traV2*		13173..13916	-		PP1Y_AT15269
**Similarity 6**	SGRAN_2844	3082797	3085343	+	*traC4*		10627..13173	-		PP1Y_AT15249
	SGRAN_2845	3085376	3085771	+			10199..10594	-		PP1Y_AT15244
	SGRAN_2846	3085768	3086175	+			9795..10202	-		PP1Y_AT15240
	SGRAN_2847	3086162	3086686	+	*trbI6*		9284..9808	-		PP1Y_AT15235
	SGRAN_2848	3086683	3087327	+	*traW*		8643..9242	-		PP1Y_AT15227
	SGRAN_2849	3087324	3088352	+	*traU*		7618..8646	-		PP1Y_AT15215
	SGRAN_2850	3088403	3089113	+	*trbC5*		6857..7621	-		PP1Y_AT15208
	SGRAN_2851	3089110	3090831	+	*traN*		5139..6860	-		PP1Y_AT15195
	SGRAN_2852	3090812	3091648	+	*traF5*		4322..5158	-		PP1Y_AT15185
	SGRAN_2853	3091638	3093074	+	*traH4*		2896..4320	-		PP1Y_AT15172
	SGRAN_2854	3093114	3095816	+	*traG6*		154..2856	-		PP1Y_AT15148
			3095970				1..144	+		PP1Y_AT15144
