# Supplementary material for: Genomic analysis of the nitrate-respiring Sphingopyxis granuli (formerly Sphingomonas macrogoltabida) strain TFA
Source: BMC Genomics. 2016 Feb 4;17:93. doi: 10.1186/s12864-016-2411-1 (PMC4741004; doi:10.1186/s12864-016-2411-1)
Supplement: Additional file 5: — Genes present in predicted Genomic Islands (GI) in the TFA genome. Highlighted in red and bold are those genes predicted in GIs by both SIGI-HMM and IslandPath methods. (PDF 48 kb) [file 12864_2016_2411_MOESM5_ESM.pdf]

Regions predicted as GI by IslandPath-DIMOB method.

Island nº	Island start	Island end	Length	Gene ID	Locus	Gene start	Gene end	Strand	Product
1	549355	560431	11076	citE	SGRAN_0512	548474	549358	+	Citrate lyase
					SGRAN_0513	549355	550770	+	Aldehyde dehydrogenase
					**SGRAN_0514**	**550787**	**551590**	**+**	**Uncharacterized protein**
					**SGRAN_0515**	**551602**	**552756**	**+**	**Putative epoxide hydrolase**
					**SGRAN_0516**	**552995**	**555370**	**+**	**TonB-dependent receptor**
				istA2	SGRAN_0517	555704	557194	+	IS21 family transposase
				istB	SGRAN_0518	557191	557958	+	IstB domain protein ATP-binding protein
					SGRAN_0519	557998	558381	-	Uncharacterized protein
					SGRAN_0520	558920	559267	+	Regulatory protein MarR
					SGRAN_0521	559451	560431	+	Virulence-associated protein E
2	1691609	1760539	68930	**intB**	**SGRAN_1557**	**1691609**	**1692832**	**+**	**Integrase**
					**SGRAN_1558**	**1692969**	**1693163**	**-**	**Uncharacterized protein**
				**yci**	**SGRAN_1559**	**1693154**	**1693705**	**-**	**Nuclease**
				**yjbJ**	**SGRAN_1560**	**1693770**	**1694549**	**-**	**Lytic transglycosylase**
					**SGRAN_1561**	**1694701**	**1695192**	**+**	**Uncharacterized protein**
					**SGRAN_1562**	**1695155**	**1695400**	**+**	**Phage transcriptional regulator, AlpA**
					**SGRAN_1563**	**1695610**	**1696170**	**+**	**Uncharacterized protein**
				**alkK3**	**SGRAN_1566**	**1698491**	**1699081**	**+**	**Long-chain fatty acid--CoA ligase**
					**SGRAN_1567**	**1699284**	**1699451**	**+**	**Transposase**
					**SGRAN_1568**	**1699502**	**1700356**	**+**	**IS511, transposase OrfB**
					**SGRAN_1569**	**1700512**	**1700925**	**-**	**Thioesterase**
				**bpoA**	**SGRAN_1570**	**1700922**	**1701809**	**-**	**Peroxidase**
					**SGRAN_1571**	**1701886**	**1702362**	**+**	**Marr family regulator**
					**SGRAN_1572**	**1703154**	**1704740**	**+**	**Ubiquinone biosynthesis protein**
					**SGRAN_1573**	**1704818**	**1705588**	**+**	**NUDIX hydrolase**
					**SGRAN_1574**	**1706712**	**1707623**	**+**	**NADPH:quinone oxidoreductase**
					**SGRAN_1575**	**1707747**	**1708031**	**+**	**Extradiol ring-cleavage dioxygenase**
					**SGRAN_1576**	**1708089**	**1708907**	**+**	**Protocatechuate 3,4-dioxygenase**
				**hcaE**	**SGRAN_1577**	**1708939**	**1710258**	**+**	**Ring-hydroxylating dioxygenase large subunit**
				**hcaF**	**SGRAN_1578**	**1710277**	**1710822**	**+**	**3-phenylpropionate dioxygenase subunit beta**
				**mhpE**	**SGRAN_1579**	**1711370**	**1712413**	**-**	**4-hydroxy-2-keto-pentanoic acid aldolase**
				**mhpF**	**SGRAN_1580**	**1712410**	**1713327**	**-**	**Acetaldehyde dehydrogenase 2/3**
				**mhpD**	**SGRAN_1581**	**1713406**	**1714059**	**-**	**2-keto-4-pentenoate hydratase**
					**SGRAN_1582**	**1714439**	**1715236**	**+**	**Pca regulon regulatory protein**
				**bphB**	**SGRAN_1583**	**1715247**	**1716035**	**-**	**2,3-dihydroxy-2,3-dihydrophenylpropionate dehydrogenase**
				**xylF**	**SGRAN_1584**	**1716166**	**1717008**	**+**	**2-hydroxy-6-oxo-2,4-heptadienoate hydrolase**
					**SGRAN_1585**	**1717386**	**1718666**	**+**	**AMP-dependent synthetase and ligase**
					**SGRAN_1586**	**1718714**	**1720900**	**+**	**TonB-dependent receptor**
				**fdr**	**SGRAN_1587**	**1721204**	**1722457**	**+**	**Ferredoxin--NAD(P)(+) reductase fdr**
					SGRAN_1588	1723074	1723172	+	putative insertion sequence transposase protein, IS3/IS911 family
				tISRso16b	SGRAN_1589	1723390	1724022	+	Transposase
					SGRAN_1590	1725791	1725967	-	Mobile element protein
					SGRAN_1591	1726820	1727029	+	Integrase catalytic region
				insF	SGRAN_1592	1727499	1728323	+	Transposase
					SGRAN_1593	1728811	1731543	-	TonB-dependent receptor
					SGRAN_1594	1731710	1732780	-	Acyl-CoA dehydrogenase family protein
					SGRAN_1595	1732799	1734010	-	Acyl-CoA dehydrogenase family protein
				**fdxB**	**SGRAN_1596**	**1734079**	**1734405**	**-**	**2Fe-2S ferredoxin**
				**p450**	**SGRAN_1597**	**1734434**	**1735696**	**-**	**Cytochrome P450**
					**SGRAN_1598**	**1736009**	**1736626**	**+**	**Regulatory protein GntR, HTH**
					**SGRAN_1599**	**1736677**	**1737819**	**+**	**Oxidoreductase, 2-nitropropane dioxygenase family protein**

					SGRAN_1600	1737838	1738593	+	Uncharacterized protein
					SGRAN_1601	1738590	1739762	+	Putative nonspecific lipid-transfer protein
					SGRAN_1602	1739759	1740235	+	Putative nucleic-acid-binding protein containing a Zn-ribbon
					SGRAN_1603	1740242	1741492	+	Putative acyl-CoA dehydrogenase family protein
					SGRAN_1604	1741492	1742583	+	Putative acyl-CoA dehydrogenase
				fadD19	SGRAN_1605	1742647	1744257	+	3-oxocholest-4-en-26-oate--CoA ligase
				pamO3	SGRAN_1606	1744254	1745891	+	Cyclohexanone monooxygenase
					SGRAN_1607	1745888	1746859	+	Hydrolase, putative
					SGRAN_1608	1747279	1750215	+	TonB-dependent receptor
				oprN2	SGRAN_1609	1750656	1752173	-	RND transporter
				rmrB	SGRAN_1610	1752181	1753734	-	DSBA oxidoreductase
				rmrA	SGRAN_1611	1753738	1754865	-	Multidrug ABC transporter permease
					SGRAN_1612	1755493	1756443	+	ABC-type transport system periplasmic component
					SGRAN_1613	1759940	1760539	+	Uncharacterized protein
3	1877102	1886676	9574		SGRAN_1712	1877102	1877854	+	Alpha/beta hydrolase fold protein
					SGRAN_1713	1878010	1878573	+	Uncharacterized protein
				int	SGRAN_1715	1878882	1880135	+	Integrase
				yjbJ2	SGRAN_1716	1880361	1881020	-	Lytic transglycosylase
					SGRAN_1717	1881148	1882056	+	Uncharacterized protein
					SGRAN_1718	1882160	1884628	+	Putative methylase/helicase
					SGRAN_1719	1884767	1885771	-	Phage integrase
					SGRAN_1720	1885768	1886682	-	Phage integrase
4	2001817	2012061	10244		SGRAN_1834	2001814	2002422	-	Uncharacterized protein
					SGRAN_1835	2002422	2003687	-	Uncharacterized protein
					SGRAN_1836	2003698	2004009	-	HNH endonuclease
					SGRAN_1837	2004006	2005517	-	Terminase
					SGRAN_1838	2008279	2009439	+	Integrase
					SGRAN_1839	2009500	2011173	-	Resolvase domain
					SGRAN_1840	2011170	2011568	-	Putative transcriptional regulator
					SGRAN_1841	2011588	2011908	-	hypothetical protein
5	3066560	3103335	36775		SGRAN_2824	3065988	3066563	+	Uncharacterized protein
					SGRAN_2825	3066560	3067045	+	Uncharacterized protein
					SGRAN_2826	3067114	3067398	+	Uncharacterized protein
					SGRAN_2827	3067401	3067787	+	Uncharacterized protein
					SGRAN_2828	3067886	3068272	-	Uncharacterized protein
				cusA2	SGRAN_2829	3068251	3071415	-	Cation transporter
				cusB2	SGRAN_2830	3071412	3072917	-	Cation efflux system protein CusB
					SGRAN_2831	3072914	3074140	-	Heavy metal RND transporter
					SGRAN_2832	3074694	3075314	-	Methyltransferase type 12
					SGRAN_2834	3075913	3076545	+	Cation transporter
					SGRAN_2835	3076722	3076973	-	Putative transcriptional regulator
					SGRAN_2836	3077023	3077484	-	Uncharacterized protein
					SGRAN_2837	3077770	3078177	+	Membrane protein
				traL2	SGRAN_2838	3078204	3078491	+	Conjugal transfer pilus assembly protein TraL
				traE2	SGRAN_2839	3078504	3079076	+	Conjugal transfer pilus assembly protein TraE
				traK2	SGRAN_2840	3079076	3079834	+	Conjugal transfer protein TraK
				traB2	SGRAN_2841	3079827	3081161	+	Conjugal transfer protein TraB
				dsbC	SGRAN_2842	3081158	3082048	+	Thiol:disulfide interchange protein DsbC
				traV2	SGRAN_2843	3082054	3082797	+	Conjugal transfer pilus assembly protein TraV
				traC4	SGRAN_2844	3082797	3085343	+	Conjugal transfer protein TraC
					SGRAN_2845	3085376	3085771	+	Conjugative transfer protein
					SGRAN_2846	3085768	3086175	+	Uncharacterized protein
				trbI6	SGRAN_2847	3086162	3086686	+	Conjugal transfer pilin signal peptidase TrbI

				traW	SGRAN_2848	3086683	3087327	+	Conjugal transfer pilus assembly protein TraW
				traU	SGRAN_2849	3087324	3088352	+	Conjugal transfer protein
				trbC5	SGRAN_2850	3088403	3089113	+	Conjugal transfer pilus assembly protein TrbC
				traN	SGRAN_2851	3089110	3090831	+	Conjugal transfer mating pair stabilization protein TraN
				traF5	SGRAN_2852	3090812	3091648	+	Conjugal transfer pilus assembly protein TraF
				traH4	SGRAN_2853	3091638	3093074	+	Conjugal transfer protein TraH
				traG6	SGRAN_2854	3093114	3095816	+	Conjugal transfer protein TraG
					SGRAN_2855	3096110	3097906	-	Superfamily I DNA/RNA helicase
					SGRAN_2856	3097918	3098637	-	Uncharacterized protein
					SGRAN_2857	3098624	3100627	-	Alkaline phosphatase
					SGRAN_2858	3100624	3102345	-	Helicase
					SGRAN_2859	3102572	3102991	+	Putative transposase
				tnpB3	SGRAN_2860	3102988	3103335	+	Isocitrate lyase
6	3109158	3161042	51884	vrlL	SGRAN_2866	3109155	3110330	-	DNA methylase
					SGRAN_2867	3111067	3111393	+	Transposase IS3/IS911 family protein
				tra5	SGRAN_2868	3111390	3112295	+	Trnasposase
				vrlL2	SGRAN_2869	3112297	3113247	-	DNA methylase
					SGRAN_2870	3113278	3117003	-	ATPase
					SGRAN_2871	3117031	3117489	-	tRNA delta(2)-isopentenylpyrophosphate transferase
					SGRAN_2872	3117482	3118378	-	Transcriptional regulator
					SGRAN_2873	3118788	3119432	+	Putative phage repressor
					SGRAN_2874	3119533	3120168	+	Uncharacterized protein
					SGRAN_2875	3120168	3120365	+	Uncharacterized protein
				traI2	SGRAN_2876	3120711	3123647	-	Multifunctional conjugation protein TraI
				traD3	SGRAN_2877	3123685	3126069	-	Type IV secretory pathway VirD4 components-like protein
					SGRAN_2878	3126059	3126391	-	Uncharacterized protein
					SGRAN_2879	3126831	3127025	+	Uncharacterized protein
					SGRAN_2880	3127128	3127364	-	Uncharacterized protein
					SGRAN_2881	3127688	3127918	+	Uncharacterized protein
				ssb	SGRAN_2882	3128033	3128422	-	Single-stranded DNA-binding protein
					SGRAN_2883	3128726	3129154	-	Uncharacterized protein
					SGRAN_2884	3129165	3129626	-	Uncharacterized protein
					SGRAN_2885	3129748	3130701	-	Chromosome segregation ATPase
					SGRAN_2886	3131007	3131429	-	Uncharacterized protein
				ardC5	SGRAN_2887	3131441	3131737	-	Antirestriction protein ArdC
				ardC6	SGRAN_2888	3131734	3132636	-	Antirestriction protein
					SGRAN_2889	3132694	3134901	-	Phage integrase family protein
					SGRAN_2890	3134898	3136685	-	Uncharacterized protein
					SGRAN_2891	3136682	3138073	-	Putative phage type integrase/recombinase
				ardC7	SGRAN_2892	3138227	3138976	-	Antirestriction protein
					SGRAN_2893	3139144	3141132	-	ParB-like partition protein
					SGRAN_2894	3141481	3141777	-	Uncharacterized protein
					SGRAN_2895	3141791	3144400	-	DEAD/DEAH box helicase
					SGRAN_2896	3144397	3145359	-	hypothetical protein
					SGRAN_2897	3145622	3146134	+	Uncharacterized protein
					SGRAN_2898	3146200	3146934	-	RES domain protein
					SGRAN_2899	3146931	3147266	-	Uncharacterized protein
					SGRAN_2900	3147398	3149245	-	Putative methylase/helicase
					SGRAN_2901	3149295	3150299	-	Phage integrase
					SGRAN_2902	3150296	3151210	-	Phage integrase
					SGRAN_2903	3151204	3152469	-	Phage integrase
					SGRAN_2904	3153308	3154312	-	Integrase/recombinase
				tnp	SGRAN_2905	3154309	3155664	-	Integrase/recombinase

					**SGRAN_2906**	**3155661**	**3156893**	**-**	**Putative integrase/recombinase y4gC**
					**SGRAN_2907**	**3156984**	**3159200**	**-**	**Putative methylase/helicase**
					**SGRAN_2908**	**3159242**	**3160171**	**-**	**Virulence-associated protein E**
				**yjbJ4**	**SGRAN_2909**	**3160296**	**3160952**	**+**	**Lytic transglycosylase**

Regions predicted as GI by SIGI-HMM method.

Island nº	Island start	Island end	Length	Gene ID	Locus	Gene start	Gene end	Strand	Product
1	1896	9255	7359		SGRAN_0002	1896	2861	-	Uncharacterized protein
					SGRAN_0003	3131	3931	+	Uncharacterized protein
					SGRAN_0004	4708	5349	+	Uncharacterized protein
					SGRAN_0005	5349	7079	+	Asparagine synthase
					SGRAN_0006	7069	9255	-	Dipeptidyl aminopeptidases/acylaminoacyl-peptidases-like protein
2	42649	48748	6099		SGRAN_0037	42649	42999	+	Uncharacterized protein
					SGRAN_0038	43478	44722	+	Uncharacterized protein
					SGRAN_0039	44715	45341	+	dCTP deaminase
					SGRAN_0040	45419	45733	-	Uncharacterized protein
					SGRAN_0041	45834	46487	+	Phospholipase/Carboxylesterase
				czcD	SGRAN_0042	47123	48094	-	Cation diffusion facilitator family transporter
					SGRAN_0043	48497	48748	-	Uncharacterized protein
3	129619	134249	4630		SGRAN_0118	129619	129885	-	Uncharacterized protein
					SGRAN_0119	129949	130386	-	Uncharacterized protein
					SGRAN_0120	130622	132697	+	TonB-dependent receptor
				copM2	SGRAN_0121	132886	133515	-	Cytochrome c family protein
					SGRAN_0122	133773	134249	-	Uncharacterized protein
4	159051	176784	17733	hutC2	SGRAN_0146	159051	159812	-	Histidine utilization repressor
					SGRAN_0147	160028	161365	-	Phage integrase family protein
					SGRAN_0149	162074	162604	-	Uncharacterized protein
					SGRAN_0150	162683	163750	-	hypothetical protein
					SGRAN_0151	163747	166371	-	Uncharacterized protein
					SGRAN_0152	166437	167561	-	Uncharacterized protein
					SGRAN_0153	167684	168034	+	Heat shock protein Hsp20
					SGRAN_0154	168110	168868	+	ETC complex I subunit region
					SGRAN_0155	169553	169825	+	Uncharacterized protein
					SGRAN_0156	169844	170083	-	Hydratase/decarboxylase family protein
					SGRAN_0157	170940	172085	+	Carbamoyl-phosphate synthase, large subunit family protein
					SGRAN_0158	172150	172461	+	Uncharacterized protein
				dapA	SGRAN_0159	172869	173612	+	4-hydroxy-tetrahydrodipicolinate synthase
					SGRAN_0160	173796	173990	+	Uncharacterized protein
					SGRAN_0161	174045	174290	+	Uncharacterized protein
				kdpA	SGRAN_0162	174896	176599	+	Potassium-transporting ATPase A chain
					SGRAN_0163	176608	176784	+	Uncharacterized protein
5	178969	189781	10812	kdpC	SGRAN_0165	178969	179541	+	Potassium-transporting ATPase C chain
				kdpD	SGRAN_0166	179600	182305	+	Putative sensor protein KdpD
				kdpE	SGRAN_0167	182302	182997	+	KDP operon transcriptional regulatory protein KdpE
				ptsN2	SGRAN_0168	183045	183497	+	Nitrogen regulatory protein PtsN
				ibpA	SGRAN_0169	183494	183883	+	Molecular chaperone Hsp20
					SGRAN_0170	184317	185108	+	Transcriptional regulator, LysR family
					SGRAN_0171	185041	185280	-	Uncharacterized protein
				trbI1	SGRAN_0172	185282	186466	-	Conjugal transfer protein TrbI
				trbG1	SGRAN_0173	186463	187446	-	Conjugal transfer protein TrbG
				trbF1	SGRAN_0174	187443	188132	-	Conjugal transfer protein TrbF
				trbL1	SGRAN_0175	188129	189493	-	Conjugal transfer protein TrbL
					SGRAN_0176	189497	189781	-	Conjugal transfer protein TrbK
					SGRAN_0207	219418	219789	-	Single-stranded DNA endonuclease

6	219786	226418	6632	ardC	SGRAN_0208	219786	220082	-	Antirepressor
				ardC1	SGRAN_0209	220079	220981	-	Antirestriction protein
					SGRAN_0210	221039	223246	-	Phage integrase family protein
					SGRAN_0211	223243	225030	-	Uncharacterized protein
					SGRAN_0212	225027	226418	-	Putative phage type integrase/recombinase
7	230013	235133	5120		SGRAN_0216	229244	230026	+	Uncharacterized protein
				hels	SGRAN_0217	230013	233531	+	DEAD/DEAH box helicase
					SGRAN_0218	233704	234558	+	Uncharacterized protein
					SGRAN_0219	234555	235133	+	Uncharacterized protein
8	519591	523649	4058	virB4	SGRAN_0480	517243	519612	-	Type VI secretion protein
					SGRAN_0481	519591	519875	-	Type IV secretion system protein B3, putative
					SGRAN_0482	519879	520208	-	Putative secreted protein
					SGRAN_0483	520403	520768	+	Type IV secretory pathway, VirB2 component (Pilin)
				istA	SGRAN_0484	521064	521432	-	Transposase
				nodT	SGRAN_0485	522222	523649	-	RND transporter
				ydhJ	SGRAN_0486	523646	524554	-	HlyD family secretion protein
9	550787	555370	4583		**SGRAN_0514**	**550787**	**551590**	**+**	**Uncharacterized protein**
					**SGRAN_0515**	**551602**	**552756**	**+**	**Putative epoxide hydrolase**
					**SGRAN_0516**	**552995**	**555370**	**+**	**TonB-dependent receptor**
10	564030	568735	4705		SGRAN_0526	564030	564860	+	PRTRC system protein B
					SGRAN_0527	564857	565516	+	PRTRC system protein A
					SGRAN_0528	565506	566264	+	Thiamine biosynthesis protein ThiF
					SGRAN_0529	566379	566654	+	Uncharacterized protein
					SGRAN_0530	566651	566935	+	Uncharacterized protein
					SGRAN_0531	566996	567475	+	Uncharacterized protein
					SGRAN_0532	567497	568735	+	Uncharacterized protein
11	574301	589855	15554		SGRAN_0534	574301	574777	+	Uncharacterized protein
					SGRAN_0535	575034	576281	+	Uncharacterized protein
					SGRAN_0536	576449	577258	+	Type I restriction endonuclease subunit M
					SGRAN_0537	577396	578091	+	Uncharacterized protein
					SGRAN_0538	578166	578813	+	Uncharacterized protein
					SGRAN_0539	578825	579286	+	Uncharacterized protein
					SGRAN_0540	579330	580085	+	Uncharacterized protein
					SGRAN_0541	580769	580963	+	Uncharacterized protein
					SGRAN_0542	581096	582577	+	Methyltransferase type 11
					SGRAN_0543	582574	583092	+	Uncharacterized protein
					SGRAN_0544	583095	583535	+	Uncharacterized protein
					SGRAN_0545	583685	585385	+	Uncharacterized protein
					SGRAN_0546	585460	586050	+	Uncharacterized protein
					SGRAN_0547	586094	586633	+	Uncharacterized protein
					SGRAN_0548	586630	586995	+	Uncharacterized protein
					SGRAN_0549	587079	587501	+	Uncharacterized protein
					SGRAN_0550	587498	587713	+	Uncharacterized protein
					SGRAN_0551	587781	587969	+	Uncharacterized protein
					SGRAN_0552	587998	588192	+	Uncharacterized protein
					SGRAN_0553	588189	588737	+	Uncharacterized protein
					SGRAN_0554	589454	589855	+	Uncharacterized protein
12	593196	599665	6469		SGRAN_0559	593196	593372	-	Uncharacterized protein
					SGRAN_0560	593607	594209	+	Uncharacterized protein
					SGRAN_0561	594297	594611	+	Prevent-host-death protein
				vapC	SGRAN_0562	594608	595060	+	Ribonuclease VapC
					SGRAN_0563	595526	595921	-	Uncharacterized protein
					SGRAN_0564	596020	596256	+	Uncharacterized protein

					SGRAN_0565	596831	597304	+	Uncharacterized protein
					SGRAN_0566	597388	598413	+	Uncharacterized protein
					SGRAN_0567	598578	598901	-	Uncharacterized protein
					SGRAN_0568	599366	599665	+	CopG family transcriptional regulator
13	604988	610389	5401		SGRAN_0571	604988	605578	+	Restriction endonuclease
					SGRAN_0572	605593	606132	+	Lytic transglycosylase catalytic
				ppsA	SGRAN_0573	606698	609079	+	PEP synthase
					SGRAN_0574	609376	610389	+	UspA domain-containing protein
14	619606	627568	7962		SGRAN_0577	619606	621066	-	Aminoglycoside phosphotransferase
				hspC2	SGRAN_0578	621167	621661	-	HspC2 heat shock protein
				fixL	SGRAN_0579	622201	624024	+	Sensor protein FixL
				fixJ	SGRAN_0580	624014	624628	+	Transcriptional regulatory protein FixJ
					SGRAN_0581	624691	625122	+	Putative response regulator
					SGRAN_0582	625170	625580	+	Globin-like protein
				adhA	SGRAN_0583	625612	626619	+	Zinc-binding alcohol dehydrogenase family protein
					SGRAN_0584	626735	627568	+	UspA domain-containing protein
15	684134	688268	4134		SGRAN_0639	684134	684823	-	Cytochrome B561
				pcl	SGRAN_0640	684896	685597	-	Membrane protein
					SGRAN_0641	685791	686042	-	Uncharacterized protein
					SGRAN_0642	686537	687187	-	Uncharacterized protein
					SGRAN_0643	687309	688268	-	ErfK/YbiS/YcfS/YnhG family protein
16	707273	711582	4309		SGRAN_0659	707273	708064	+	Putative LuxR family transcriptional regulator
					SGRAN_0660	708258	709043	+	Protein of unknwon function (DUF2893)
				ync	SGRAN_0661	709048	709968	+	Uncharacterized protein (Ync)
					SGRAN_0662	710182	710535	-	Uncharacterized protein
					SGRAN_0663	711133	711582	+	Uncharacterized protein
				traG2	SGRAN_0664	711579	715349	-	DNA transfer protein
17	1432460	1447308	14848	sulII	SGRAN_1316	1432460	1433275	+	Dihydropteroate synthase type-2
				glmM	SGRAN_1317	1433362	1433664	+	Phosphoglucosamine mutase
				insA	SGRAN_1318	1433840	1435333	-	Transposase InsA
					SGRAN_1319	1435544	1435768	-	Uncharacterized protein
				parA2	SGRAN_1320	1435765	1436502	-	Plasmid partitioning protein ParA
				arsR3	SGRAN_1321	1436650	1436982	+	ArsR family transcriptional regulator
				arsH2	SGRAN_1322	1436979	1437746	+	NADPH-dependent FMN reductase ArsH
				arsC3	SGRAN_1323	1437743	1438249	+	Arsenate reductase
				arsB2	SGRAN_1324	1438246	1439313	+	Arsenical-resistance protein
					SGRAN_1325	1439428	1439631	+	Putative transcriptional regulator
				trbL2	SGRAN_1327	1440209	1441636	-	Conjugal transfer protein TrbL
					SGRAN_1328	1441647	1441883	-	Entry exclusion lipoprotein TrbK
				trbJ2	SGRAN_1329	1441896	1442675	-	Conjugal transfer protein TrbJ
				repC	SGRAN_1331	1444037	1444918	-	Replication protein C
				repA2	SGRAN_1332	1444905	1445732	-	RepA replication protein
					SGRAN_1333	1446106	1447308	-	Integrase
18	1560087	1565535	5448		SGRAN_1435	1560087	1560917	+	Uncharacterized protein
					SGRAN_1436	1560927	1563416	+	p pilus assembly protein, porin PapC
					SGRAN_1437	1563413	1564300	+	Uncharacterized protein
					SGRAN_1438	1564834	1565535	-	Uncharacterized protein
				**intB**	**SGRAN_1557**	**1691609**	**1692832**	**+**	**Integrase**
					**SGRAN_1558**	**1692969**	**1693163**	**-**	**Uncharacterized protein**
				**yci**	**SGRAN_1559**	**1693154**	**1693705**	**-**	**Nuclease**
				**yjbJ**	**SGRAN_1560**	**1693770**	**1694549**	**-**	**Lytic transglycosylase**
					**SGRAN_1561**	**1694701**	**1695192**	**+**	**Uncharacterized protein**
					**SGRAN_1562**	**1695155**	**1695400**	**+**	**Phage transcriptional regulator, AlpA**

19	1691609	1722457	30848		SGRAN_1563	1695610	1696170	+	Uncharacterized protein
				alkK3	SGRAN_1566	1698491	1699081	+	Long-chain fatty acid--CoA ligase
					SGRAN_1567	1699284	1699451	+	Transposase
					SGRAN_1568	1699502	1700356	+	IS511, transposase OrfB
					SGRAN_1569	1700512	1700925	-	Thioesterase
				bpoA	SGRAN_1570	1700922	1701809	-	Peroxidase
					SGRAN_1571	1701886	1702362	+	Marr family regulator
					SGRAN_1572	1703154	1704740	+	Ubiquinone biosynthesis protein
					SGRAN_1573	1704818	1705588	+	NUDIX hydrolase
					SGRAN_1574	1706712	1707623	+	NADPH:quinone oxidoreductase
					SGRAN_1575	1707747	1708031	+	Extradiol ring-cleavage dioxygenase
					SGRAN_1576	1708089	1708907	+	Protocatechuate 3,4-dioxygenase
				hcaE	SGRAN_1577	1708939	1710258	+	Ring-hydroxylating dioxygenase large subunit
				hcaF	SGRAN_1578	1710277	1710822	+	3-phenylpropionate dioxygenase subunit beta
				mhpE	SGRAN_1579	1711370	1712413	-	4-hydroxy-2-keto-pentanoic acid aldolase
				mhpF	SGRAN_1580	1712410	1713327	-	Acetaldehyde dehydrogenase 2/3
				mhpD	SGRAN_1581	1713406	1714059	-	2-keto-4-pentenoate hydratase
					SGRAN_1582	1714439	1715236	+	Pca regulon regulatory protein
				bphB	SGRAN_1583	1715247	1716035	-	2,3-dihydroxy-2,3-dihydrophenylpropionate dehydrogenase
				xylF	SGRAN_1584	1716166	1717008	+	2-hydroxy-6-oxo-2,4-heptadienoate hydrolase
					SGRAN_1585	1717386	1718666	+	AMP-dependent synthetase and ligase
					SGRAN_1586	1718714	1720900	+	TonB-dependent receptor
				fdr	SGRAN_1587	1721204	1722457	+	Ferredoxin--NAD(P)(+) reductase fdr
20	1723390	1731543	8153	tISRso16b	SGRAN_1589	1723390	1724022	+	Transposase
					SGRAN_1590	1725791	1725967	-	Mobile element protein
					SGRAN_1591	1726820	1727029	+	Integrase catalytic region
				insF	SGRAN_1592	1727499	1728323	+	Transposase
					SGRAN_1593	1728811	1731543	-	TonB-dependent receptor
21	1734079	1761611	27532	fdxB	SGRAN_1596	1734079	1734405	-	2Fe-2S ferredoxin
				p450	SGRAN_1597	1734434	1735696	-	Cytochrome P450
					SGRAN_1598	1736009	1736626	+	Regulatory protein GntR, HTH
					SGRAN_1599	1736677	1737819	+	Oxidoreductase, 2-nitropropane dioxygenase family protein
					SGRAN_1600	1737838	1738593	+	Uncharacterized protein
					SGRAN_1601	1738590	1739762	+	Putative nonspecific lipid-transfer protein
					SGRAN_1602	1739759	1740235	+	Putative nucleic-acid-binding protein containing a Zn-ribbon
					SGRAN_1603	1740242	1741492	+	Putative acyl-CoA dehydrogenase family protein
					SGRAN_1604	1741492	1742583	+	Putative acyl-CoA dehydrogenase
				fadD19	SGRAN_1605	1742647	1744257	+	3-oxocholest-4-en-26-oate--CoA ligase
				pamO3	SGRAN_1606	1744254	1745891	+	Cyclohexanone monooxygenase
					SGRAN_1607	1745888	1746859	+	Hydrolase, putative
					SGRAN_1608	1747279	1750215	+	TonB-dependent receptor
				oprN2	SGRAN_1609	1750656	1752173	-	RND transporter
				rmrB	SGRAN_1610	1752181	1753734	-	DSBA oxidoreductase
				rmrA	SGRAN_1611	1753738	1754865	-	Multidrug ABC transporter permease
					SGRAN_1612	1755493	1756443	+	ABC-type transport system periplasmic component
					SGRAN_1613	1759940	1760539	+	Uncharacterized protein
					SGRAN_1614	1760784	1761611	+	Putative secreted protein
22	1878882	1891309	12427	int	SGRAN_1715	1878882	1880135	+	Integrase
				yjbJ2	SGRAN_1716	1880361	1881020	-	Lytic transglycosylase
					SGRAN_1717	1881148	1882056	+	Uncharacterized protein
					SGRAN_1718	1882160	1884628	+	Putative methylase/helicase
					SGRAN_1719	1884767	1885771	-	Phage integrase
					SGRAN_1720	1885768	1886682	-	Phage integrase

					SGRAN_1721	1886676	1887941	-	Integrase
					SGRAN_1722	1888280	1890124	+	Putative methylase/helicase
					SGRAN_1723	1890258	1890593	+	Uncharacterized protein
					SGRAN_1724	1890590	1891309	+	RES domain protein
23	1919110	1924114	5004		SGRAN_1751	1919110	1919559	-	Uncharacterized protein
					SGRAN_1752	1919688	1920626	-	Thioredoxin-like protein
				parD	SGRAN_1753	1920899	1921123	+	Addiction module antitoxin
					SGRAN_1754	1921120	1921449	+	Plasmid stabilization protein
					SGRAN_1755	1921512	1921676	-	Type IV secretory protease
					SGRAN_1756	1921828	1922133	+	hypothetical protein
					SGRAN_1757	1922240	1923286	+	Competence protein
					SGRAN_1758	1923374	1924114	+	Uncharacterized protein
24	2000239	2009439	9200		SGRAN_1832	2000239	2001300	-	Uncharacterized protein
					SGRAN_1833	2001302	2001817	-	Uncharacterized protein
					**SGRAN_1834**	**2001814**	**2002422**	**-**	**Uncharacterized protein**
					**SGRAN_1835**	**2002422**	**2003687**	**-**	**Uncharacterized protein**
					**SGRAN_1836**	**2003698**	**2004009**	**-**	**HNH endonuclease**
					**SGRAN_1837**	**2004006**	**2005517**	**-**	**Terminase**
					**SGRAN_1838**	**2008279**	**2009439**	**+**	**Integrase**
25	2025209	2032500	7291		SGRAN_1857	2025209	2025451	+	Helix-turn-helix domain protein
				lysR2	SGRAN_1858	2025434	2026393	-	LysR family transcriptional regulator
				hyl1	SGRAN_1859	2026628	2027641	-	Epoxide hydrolase domain protein
					SGRAN_1860	2027855	2028748	+	LysR family transcriptional regulator
					SGRAN_1861	2028745	2029524	-	AraC-type DNA-binding domain-containing protein
					SGRAN_1862	2029623	2030414	+	inositol-1-monophosphatase
					SGRAN_1863	2030923	2031846	+	Oxidoreductase
					SGRAN_1864	2031880	2032500	-	Pentapeptide repeat protein
26	2048540	2055671	7131		SGRAN_1884	2048540	2048947	-	Uncharacterized protein
					SGRAN_1885	2049437	2050462	+	Filamentation induced by cAMP protein Fic
					SGRAN_1886	2050474	2052357	+	Sir2-family regulator
					SGRAN_1887	2052357	2053805	+	Nuclease PIN
					SGRAN_1888	2053929	2055671	-	Retron-type RNA-directed DNA polymerase
27	2442400	2447644	5244		SGRAN_2245	2442400	2446809	-	ATPase
					SGRAN_2246	2447063	2447644	-	Uncharacterized protein
					SGRAN_2247	2447641	2448345	-	hypothetical protein
28	2569535	2589714	20179	wecA	SGRAN_2366	2569535	2570878	-	UDP-N-acetylgalactosamine-undecaprenyl-phosphate N-acetylgalactosaminephosphotransferase
				pglA	SGRAN_2367	2571046	2572257	-	Glycosyl transferase family 1
				asnB	SGRAN_2368	2572342	2574279	-	Asparagine synthase
					SGRAN_2369	2574279	2575358	-	Glycosyl transferase group 1
					SGRAN_2370	2575521	2576450	-	Uncharacterized protein
					SGRAN_2371	2576500	2577630	+	Glycosyltransferase
					SGRAN_2372	2577606	2578529	-	Glycosyl transferase
					SGRAN_2373	2578532	2579365	-	FkbM family methyltransferase
					SGRAN_2374	2579362	2580639	-	Uncharacterized protein
				wbpO	SGRAN_2375	2580722	2582035	-	Vi polysaccharide biosynthesis protein VipA/TviB
				lspL	SGRAN_2376	2582032	2583051	-	UDP-glucuronate 5-epimerase
					SGRAN_2377	2583072	2584328	-	Uncharacterized protein
					SGRAN_2378	2584356	2586566	-	Non-specific protein-tyrosine kinase
					SGRAN_2379	2586583	2587320	-	Polysaccharide export protein
					SGRAN_2380	2587382	2588836	-	O-antigen polymerase
					SGRAN_2381	2588938	2589714	-	Metallophosphoesterase
				thnM	SGRAN_2788	3027481	3029652	-	TonB-dependent receptor-like protein
				thnL	SGRAN_2789	3030049	3032136	-	Enoyl-CoA hydratase/3-hydroxyacyl-CoA dehydrogenase-like protein

29	3027481	3038527	11046	thnK	SGRAN_2790	3032129	3033268	-	Pimeloyl-CoA dehydrogenase small subunit-like protein
				thnJ	SGRAN_2791	3033271	3034464	-	Pimeloyl-CoA dehydrogenase large subunit-like protein
				thnI	SGRAN_2792	3034480	3035688	-	Acetyl-CoA acteyl transferase-like protein
				thnH	SGRAN_2793	3035693	3036910	-	CoA-transferase-like protein
				thnG	SGRAN_2794	3037100	3038527	-	Non-acylating NAD-dependent aldehyde dehydrogenase
30	3043041	3060895	17854	thnB	SGRAN_2800	3043041	3043853	-	Cis-dihydrodiol dehydrogenase
				thnC	SGRAN_2801	3044247	3045161	+	1,2-dihydroxynaphthalene dioxygenase ThnC
				thnA3	SGRAN_2802	3045267	3045581	+	Rieske-type ferredoxin
				thnA4	SGRAN_2803	3045585	3046604	+	Ferredoxin reductase
				thnR	SGRAN_2804	3046775	3047701	+	LysR-type activator
				thnY	SGRAN_2805	3047892	3048683	+	Ferredoxin reductase-like
				copB2	SGRAN_2806	3050047	3050715	-	Copper resistance protein B
				copA5	SGRAN_2807	3051102	3052739	-	Copper resistance protein A
					SGRAN_2808	3052952	3053389	-	RNA polymerase
					SGRAN_2809	3053519	3053959	-	Heavy metal resistance protein
					SGRAN_2810	3053956	3054240	-	Uncharacterized protein
					SGRAN_2811	3054340	3055038	+	Uncharacterized protein
					SGRAN_2812	3055436	3055903	+	Metal-binding protein
					SGRAN_2813	3055924	3056307	+	Uncharacterized protein
					SGRAN_2814	3056353	3056625	-	Copper-sensing transcriptional repressor CsoR family protein
					SGRAN_2815	3056747	3057013	+	Uncharacterized protein
					SGRAN_2816	3057073	3058455	+	mRNA 3'-end processing factor
				phnN	SGRAN_2817	3058452	3059963	+	Putative thymidine phosphorylase
				prs1	SGRAN_2818	3059960	3060895	+	Ribose-phosphate pyrophosphokinase
				copA6	SGRAN_2819	3060892	3063261	+	Cu(+)-exporting ATPase
31	3063300	3079834	16534		SGRAN_2820	3063300	3063539	+	Membrane protein
					SGRAN_2821	3063536	3064180	+	Isoprenylcysteine carboxyl methyltransferase
				ybaT	SGRAN_2822	3064199	3065542	+	Amino acid permease
					SGRAN_2823	3065603	3065986	+	Membrane protein
					**SGRAN_2824**	**3065988**	**3066563**	**+**	**Uncharacterized protein**
					**SGRAN_2825**	**3066560**	**3067045**	**+**	**Uncharacterized protein**
					**SGRAN_2826**	**3067114**	**3067398**	**+**	**Uncharacterized protein**
					**SGRAN_2827**	**3067401**	**3067787**	**+**	**Uncharacterized protein**
					**SGRAN_2828**	**3067886**	**3068272**	**-**	**Uncharacterized protein**
				**cusA2**	**SGRAN_2829**	**3068251**	**3071415**	**-**	**Cation transporter**
				**cusB2**	**SGRAN_2830**	**3071412**	**3072917**	**-**	**Cation efflux system protein CusB**
					**SGRAN_2831**	**3072914**	**3074140**	**-**	**Heavy metal RND transporter**
					**SGRAN_2832**	**3074694**	**3075314**	**-**	**Methyltransferase type 12**
					**SGRAN_2834**	**3075913**	**3076545**	**+**	**Cation transporter**
					**SGRAN_2835**	**3076722**	**3076973**	**-**	**Putative transcriptional regulator**
					**SGRAN_2836**	**3077023**	**3077484**	**-**	**Uncharacterized protein**
					**SGRAN_2837**	**3077770**	**3078177**	**+**	**Membrane protein**
				**traL2**	**SGRAN_2838**	**3078204**	**3078491**	**+**	**Conjugal transfer pilus assembly protein TraL**
				**traE2**	**SGRAN_2839**	**3078504**	**3079076**	**+**	**Conjugal transfer pilus assembly protein TraE**
				**traK2**	**SGRAN_2840**	**3079076**	**3079834**	**+**	**Conjugal transfer protein TraK**
				**traB2**	**SGRAN_2841**	**3079827**	**3081161**	**+**	**Conjugal transfer protein TraB**
32	3093114	3102991	9877	**traG6**	**SGRAN_2854**	**3093114**	**3095816**	**+**	**Conjugal transfer protein TraG**
					**SGRAN_2855**	**3096110**	**3097906**	**-**	**Superfamily I DNA/RNA helicase**
					**SGRAN_2856**	**3097918**	**3098637**	**-**	**Uncharacterized protein**
					**SGRAN_2857**	**3098624**	**3100627**	**-**	**Alkaline phosphatase**
					**SGRAN_2858**	**3100624**	**3102345**	**-**	**Helicase**
					**SGRAN_2859**	**3102572**	**3102991**	**+**	**Putative transposase**
				**tnpB3**	**SGRAN_2860**	**3102988**	**3103335**	**+**	**Isocitrate lyase**

33	3107612	3127918	20306		SGRAN_2864	3107612	3108181	+	Plasmid pRiA4b ORF-3-like family protein
					SGRAN_2865	3108178	3109158	-	Helicase domain protein
				**vrlL**	**SGRAN_2866**	**3109155**	**3110330**	**-**	**DNA methylase**
					**SGRAN_2867**	**3111067**	**3111393**	**+**	**Transposase IS3/IS911 family protein**
				**tra5**	**SGRAN_2868**	**3111390**	**3112295**	**+**	**Transposase**
				**vrlL2**	**SGRAN_2869**	**3112297**	**3113247**	**-**	**DNA methylase**
					**SGRAN_2870**	**3113278**	**3117003**	**-**	**ATPase**
					**SGRAN_2871**	**3117031**	**3117489**	**-**	**tRNA delta(2)-isopentenylpyrophosphate transferase**
					**SGRAN_2872**	**3117482**	**3118378**	**-**	**Transcriptional regulator**
					**SGRAN_2873**	**3118788**	**3119432**	**+**	**Putative phage repressor**
					**SGRAN_2874**	**3119533**	**3120168**	**+**	**Uncharacterized protein**
					**SGRAN_2875**	**3120168**	**3120365**	**+**	**Uncharacterized protein**
				**traI2**	**SGRAN_2876**	**3120711**	**3123647**	**-**	**Multifunctional conjugation protein TraI**
				**traD3**	**SGRAN_2877**	**3123685**	**3126069**	**-**	**Type IV secretory pathway VirD4 components-like protein**
					**SGRAN_2878**	**3126059**	**3126391**	**-**	**Uncharacterized protein**
					**SGRAN_2879**	**3126831**	**3127025**	**+**	**Uncharacterized protein**
					**SGRAN_2880**	**3127128**	**3127364**	**-**	**Uncharacterized protein**
					**SGRAN_2881**	**3127688**	**3127918**	**+**	**Uncharacterized protein**
34	3128726	3138976	10250		**SGRAN_2883**	**3128726**	**3129154**	**-**	**Uncharacterized protein**
					**SGRAN_2884**	**3129165**	**3129626**	**-**	**Uncharacterized protein**
					**SGRAN_2885**	**3129748**	**3130701**	**-**	**Chromosome segregation ATPase**
					**SGRAN_2886**	**3131007**	**3131429**	**-**	**Uncharacterized protein**
				**ardC5**	**SGRAN_2887**	**3131441**	**3131737**	**-**	**Antirestriction protein ArdC**
				**ardC6**	**SGRAN_2888**	**3131734**	**3132636**	**-**	**Antirestriction protein**
					**SGRAN_2889**	**3132694**	**3134901**	**-**	**Phage integrase family protein**
					**SGRAN_2890**	**3134898**	**3136685**	**-**	**Uncharacterized protein**
					**SGRAN_2891**	**3136682**	**3138073**	**-**	**Putative phage type integrase/recombinase**
				**ardC7**	**SGRAN_2892**	**3138227**	**3138976**	**-**	**Antirestriction protein**
35	3141481	3162313	20832		**SGRAN_2894**	**3141481**	**3141777**	**-**	**Uncharacterized protein**
					**SGRAN_2895**	**3141791**	**3144400**	**-**	**DEAD/DEAH box helicase**
					**SGRAN_2896**	**3144397**	**3145359**	**-**	**hypothetical protein**
					**SGRAN_2897**	**3145622**	**3146134**	**+**	**Uncharacterized protein**
					**SGRAN_2898**	**3146200**	**3146934**	**-**	**RES domain protein**
					**SGRAN_2899**	**3146931**	**3147266**	**-**	**Uncharacterized protein**
					**SGRAN_2900**	**3147398**	**3149245**	**-**	**Putative methylase/helicase**
					**SGRAN_2901**	**3149295**	**3150299**	**-**	**Phage integrase**
					**SGRAN_2902**	**3150296**	**3151210**	**-**	**Phage integrase**
					**SGRAN_2903**	**3151204**	**3152469**	**-**	**Phage integrase**
					**SGRAN_2904**	**3153308**	**3154312**	**-**	**Integrase/recombinase**
				**tnp**	**SGRAN_2905**	**3154309**	**3155664**	**-**	**Integrase/recombinase**
					**SGRAN_2906**	**3155661**	**3156893**	**-**	**Putative integrase/recombinase y4gC**
					**SGRAN_2907**	**3156984**	**3159200**	**-**	**Putative methylase/helicase**
					**SGRAN_2908**	**3159242**	**3160171**	**-**	**Virulence-associated protein E**
				**yjbJ4**	**SGRAN_2909**	**3160296**	**3160952**	**+**	**Lytic transglycosylase**
					SGRAN_2910	3161042	3162313	-	Phage integrase
36	3414906	3423254	8348	cas1	SGRAN_3113	3414906	3415973	-	CRISPR-associated endonuclease Cas1
					SGRAN_3114	3417028	3417597	+	Uncharacterized protein
					SGRAN_3115	3417972	3420977	+	Uncharacterized protein
				dam	SGRAN_3116	3421055	3421942	-	D12 class N6 adenine-specific DNA methyltransferase
				msi303	SGRAN_3117	3421977	3423254	-	Uncharacterized protein
37	3502309	3506773	4464		SGRAN_3197	3502309	3504747	-	Uncharacterized protein
					SGRAN_3198	3506000	3506773	+	Uncharacterized protein
					SGRAN_3207	3516294	3517106	+	Uncharacterized protein

38	3516294	3525246	8952		SGRAN_3208	3517484	3518047	+	Uncharacterized protein
					SGRAN_3209	3518264	3518971	-	Putative nucleotidyltransferase
					SGRAN_3210	3519071	3519355	+	Transposase
					SGRAN_3211	3519376	3520041	+	Transposase
					SGRAN_3212	3520002	3520244	+	Integrase
					SGRAN_3213	3520263	3520496	-	Nucleotidyltransferase
					SGRAN_3214	3520594	3520917	-	Uncharacterized protein
					SGRAN_3215	3521254	3522939	+	Uncharacterized protein
					SGRAN_3216	3522936	3524741	+	Chemotaxis protein
					SGRAN_3217	3524827	3525246	+	Putative transposase
				tnpB	SGRAN_3218	3525243	3525590	+	Isocitrate lyase
39	3540618	3545271	4653		SGRAN_3231	3540618	3541352	-	LuxR family transcriptional regulator
					SGRAN_3232	3542157	3544619	+	Uncharacterized protein
					SGRAN_3233	3544627	3545271	-	Uncharacterized protein
40	3765327	3770696	5369		SGRAN_3447	3765327	3769652	-	YD repeat-containing protein
					SGRAN_3448	3769716	3769940	-	RHS Repeat protein
					SGRAN_3449	3770108	3770497	-	Uncharacterized protein
					SGRAN_3450	3770499	3770696	-	Uncharacterized protein
41	4378659	4382908	4249		SGRAN_3981	4378659	4379792	-	Phage integrase
					SGRAN_3982	4381168	4382250	+	RNA-directed DNA polymerase
				resU	SGRAN_3983	4382231	4382908	-	Resolvase domain protein
42	4580571	4584860	4289		SGRAN_4167	4580571	4581650	+	PepSY-associated TM helix
				copB3	SGRAN_4168	4582039	4583055	-	Copper resistance protein B
				copA7	SGRAN_4169	4583052	4584860	-	Copper resistance protein A
43	4588116	4600864	12748	copA8	SGRAN_4170	4585744	4588119	+	Cu(+)-exporting ATPase
				hmrR3	SGRAN_4171	4588116	4588529	+	HTH-type transcriptional regulator HmrR
				copD	SGRAN_4172	4588754	4589674	-	Copper resistance protein D
					SGRAN_4173	4589870	4590736	-	Transposase
					SGRAN_4174	4590777	4591040	-	Transposase
				copC	SGRAN_4175	4591056	4591364	-	Copper resistance protein CopC
				copA9	SGRAN_4176	4593011	4594771	+	Copper resistance protein A
				pcoB2	SGRAN_4177	4594768	4595928	+	Copper resistance protein B
				copM	SGRAN_4178	4595963	4596550	+	Cytochrome c family protein
					SGRAN_4179	4597745	4598020	+	Uncharacterized protein
				czcC2	SGRAN_4180	4598154	4599380	+	Heavy metal RND efflux outer membrane protein, CzcC family
				cusB3	SGRAN_4181	4599377	4600864	+	Cation efflux system protein CusBr
				cusA3	SGRAN_4182	4600861	4603989	+	Cation transporter
44	4604001	4614202	10201		SGRAN_4183	4604001	4604366	+	Copper-binding protein CusF
				cnrT	SGRAN_4184	4604421	4605497	+	DMT(Drug/metabolite transporter) superfamily permease
				cadA2	SGRAN_4185	4605728	4608001	-	Heavy metal translocating P-type ATPase
				merR5	SGRAN_4186	4608069	4608488	+	MerR family transcriptional regulator
				pbrT2	SGRAN_4187	4608588	4610582	+	Iron permease
					SGRAN_4188	4610621	4610995	+	Cobalt transporter, cation efflux protein
					SGRAN_4189	4611529	4612836	+	Outer membrane efflux protein
				czcB4	SGRAN_4190	4613042	4614202	+	Metal transporter
					SGRAN_4192	4617463	4617807	+	Uncharacterized protein
				zntA	SGRAN_4193	4618613	4621102	+	Heavy metal translocating P-type ATPase
				bepC	SGRAN_4194	4621099	4622610	+	Type I secretion outer membrane protein, TolC
					SGRAN_4195	4622607	4623260	+	Uncharacterized protein
				czcD4	SGRAN_4196	4623245	4624183	+	Cadmium, cobalt and zinc/H(+)-K(+) antiporter
					SGRAN_4197	4624245	4624520	+	Uncharacterized protein
					SGRAN_4198	4624566	4625048	+	Uncharacterized protein
					SGRAN_4199	4625241	4625648	+	Uncharacterized protein

45	4617463	4638344	20881	merT	SGRAN_4200	4626988	4627383	+	Mercury transporter MerT
				merP	SGRAN_4201	4627396	4627725	+	MerP protein
				merA	SGRAN_4202	4627764	4629221	+	Hg(II) reductase
				czcA5	SGRAN_4203	4629327	4632542	-	Cation transporter
				hylD2	SGRAN_4204	4632545	4633696	-	Secretion protein HlyD
					SGRAN_4205	4633699	4634964	-	Outer membrane efflux protein
				arsH4	SGRAN_4206	4635761	4636504	-	NADPH-dependent FMN reductase ArsH
				arsB4	SGRAN_4207	4636508	4637578	-	Arsenical-resistance protein
				arsC7	SGRAN_4208	4637578	4638003	-	Arsenate reductase
				arsR7	SGRAN_4209	4638015	4638344	-	ArsR family transcriptional regulator
46	4652653	4656862	4209		SGRAN_4219	4652653	4652826	+	Uncharacterized protein
					SGRAN_4220	4653246	4653524	+	Uncharacterized protein
					SGRAN_4221	4653668	4653865	+	Uncharacterized protein
					SGRAN_4222	4653954	4654448	+	Uncharacterized protein
					SGRAN_4223	4654564	4654851	+	Transcriptional regulator
				repA4	SGRAN_4224	4654858	4655973	+	RepA replication protein
				parA4	SGRAN_4225	4655970	4656608	+	Plasmid partitioning protein ParA
					SGRAN_4226	4656605	4656862	+	Uncharacterized protein
					SGRAN_4227	4656859	4657338	+	Glycosidase
47	4674319	4679853	5534	trbI5	SGRAN_4244	4673159	4674322	+	Conjugal transfer protein TraI
					SGRAN_4245	4674319	4674540	+	Uncharacterized protein
					SGRAN_4246	4674602	4674904	-	Uncharacterized protein
					SGRAN_4247	4674891	4675205	-	Transcriptional regulator, LysR family
					SGRAN_4248	4676434	4677420	-	Uncharacterized protein
					SGRAN_4249	4678298	4679194	-	Abortive phage resistance protein
					SGRAN_4250	4679191	4679853	-	Uncharacterized protein
