# Supplementary material for: Genomic analysis of the nitrate-respiring Sphingopyxis granuli (formerly Sphingomonas macrogoltabida) strain TFA
Source: BMC Genomics. 2016 Feb 4;17:93. doi: 10.1186/s12864-016-2411-1 (PMC4741004; doi:10.1186/s12864-016-2411-1)
Supplement: Additional file 6: — Pathway Tools biodegradation pathways predicted in TFA. ID of genes involved in each reaction is shown in red. (PDF 53 kb) [file 12864_2016_2411_MOESM6_ESM.pdf]

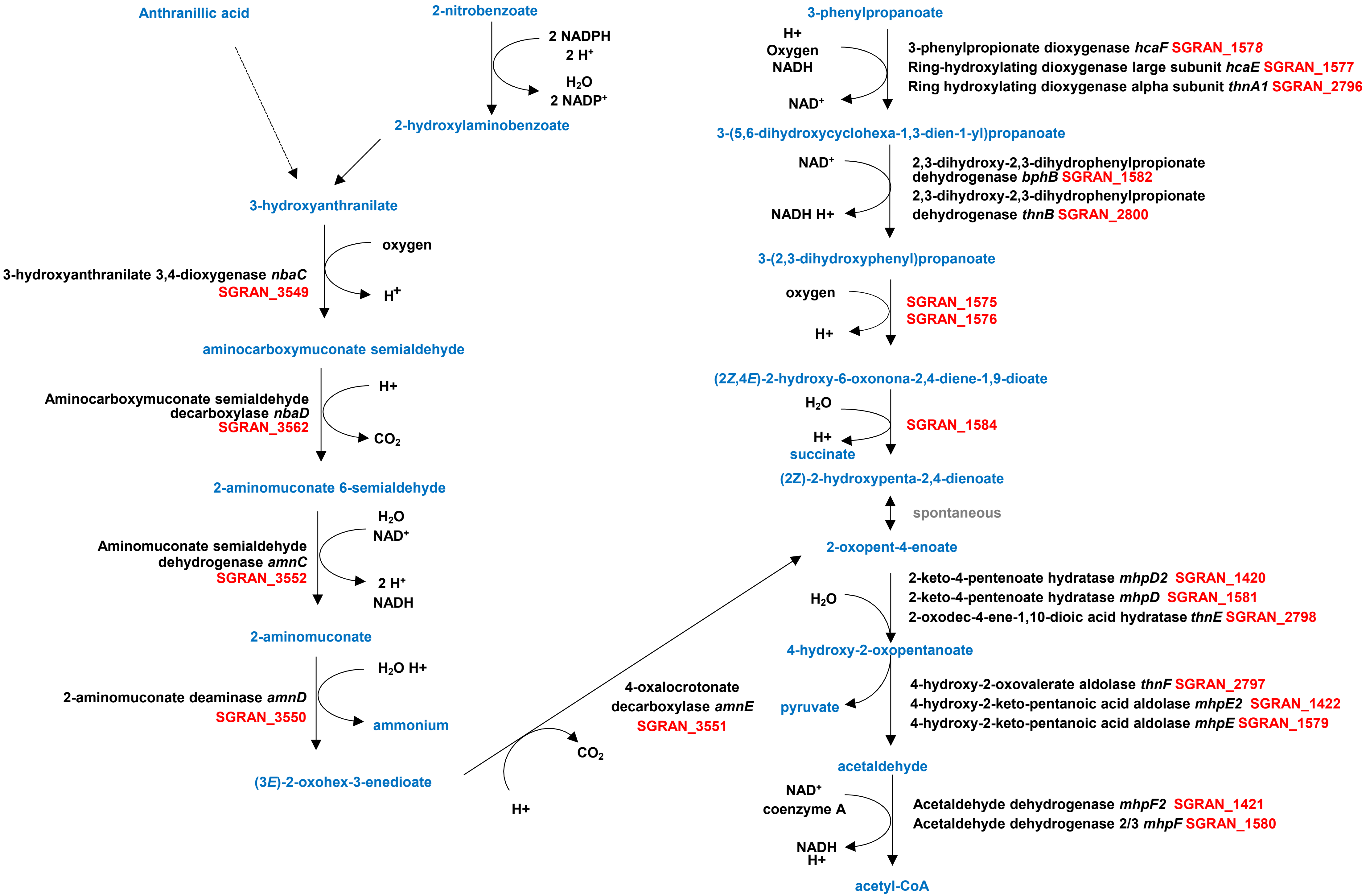
Anthranillic acid
2-nitrobenzoate
2 NADPH
2 H+
H_2O
2 NADP+
2-hydroxylaminobenzoate
3-hydroxyanthranilate
oxygen
3-hydroxyanthranilate 3,4-dioxygenase nbaC
SGRAN_3549
H+
aminocarboxymuconate semialdehyde
H+
Aminocarboxymuconate semialdehyde decarboxylase nbaD
SGRAN_3562
CO_2
2-aminomuconate 6-semialdehyde
H_2O
NAD+
Aminomuconate semialdehyde dehydrogenase amnC
SGRAN_3552
2 H+
NADH
2-aminomuconate
H_2O H+
2-aminomuconate deaminase amnD
SGRAN_3550
ammonium
(3E)-2-oxohex-3-enedioate
H+
CO_2
4-oxalocrotonate decarboxylase amnE
SGRAN_3551
3-phenylpropanoate
H+
Oxygen
NADH
NAD+
3-phenylpropionate dioxygenase hcaF SGRAN_1578
Ring-hydroxylating dioxygenase large subunit hcaE SGRAN_1577
Ring hydroxylating dioxygenase alpha subunit thnA1 SGRAN_2796
3-(5,6-dihydroxycyclohexa-1,3-dien-1-yl)propanoate
NAD+
NADH H+
2,3-dihydroxy-2,3-dihydrophenylpropionate dehydrogenase bphB SGRAN_1582
2,3-dihydroxy-2,3-dihydrophenylpropionate dehydrogenase thnB SGRAN_2800
3-(2,3-dihydroxyphenyl)propanoate
oxygen
H+
SGRAN_1575
SGRAN_1576
(2Z,4E)-2-hydroxy-6-oxonona-2,4-diene-1,9-dioate
H_2O
H+
succinate
SGRAN_1584
(2Z)-2-hydroxypenta-2,4-dienoate
spontaneous
2-oxopent-4-enoate
H_2O
2-keto-4-pentenoate hydratase mhpD2 SGRAN_1420
2-keto-4-pentenoate hydratase mhpD SGRAN_1581
2-oxodec-4-ene-1,10-dioic acid hydratase thnE SGRAN_2798
4-hydroxy-2-oxopentanoate
pyruvate
4-hydroxy-2-oxovalerate aldolase thnF SGRAN_2797
4-hydroxy-2-keto-pentanoic acid aldolase mhpE2 SGRAN_1422
4-hydroxy-2-keto-pentanoic acid aldolase mhpE SGRAN_1579
acetaldehyde
NAD+
coenzyme A
NADH
H+
Acetaldehyde dehydrogenase mhpF2 SGRAN_1421
Acetaldehyde dehydrogenase 2/3 mhpF SGRAN_1580
acetyl-CoA
